# Curcumin modulates chronic myelogenous leukemia exosomes composition and affects angiogenic phenotype *via* exosomal miR-21

**DOI:** 10.18632/oncotarget.8483

**Published:** 2016-03-30

**Authors:** Simona Taverna, Simona Fontana, Francesca Monteleone, Marzia Pucci, Laura Saieva, Viviana De Caro, Valeria Giunta Cardinale, Marco Giallombardo, Emanuela Vicario, Christian Rolfo, Giacomo De Leo, Riccardo Alessandro

**Affiliations:** ^1^ Dipartimento di Biopatologia e Metodologie Biomediche, Sezione di Biologia e Genetica, Università di Palermo, Palermo, Italy; ^2^ Dipartimento di Scienze e Tecnologie Biologiche Chimiche e Farmaceutiche, Università di Palermo, Palermo, Italy; ^3^ Phase I-Early Clinical Trials Unit, Oncology Department, Antwerp University Hospital (UZA) and Center for Oncological Research (CORE) Antwerp University, Edegem, Antwerp, Belgium

**Keywords:** exosomes, curcumin, miR-21, CML

## Abstract

Tumor derived exosomes are vesicles which contain proteins and microRNAs that mediate cell-cell communication and are involved in angiogenesis and tumor progression. Curcumin derived from the plant *Curcuma longa,* shows anticancer effects. Exosomes released by CML cells treated with Curcumin contain a high amount of miR-21 that is shuttled into the endothelial cells in a biologically active form. The treatment of HUVECs with CML Curcu-exosomes reduced RhoB expression and negatively modulated endothelial cells motility. We showed that the addition of CML control exosomes to HUVECs caused an increase in IL8 and VCAM1 levels, but Curcu-exosomes reversed these effects thus attenuating their angiogenic properties. This antiangiogenic effect was confirmed with *in vitro* and *in vivo* vascular network formation assays. SWATH analysis of the proteomic profile of Curcu-exosomes revealed that Curcumin treatment deeply changes their molecular properties, in particular, Curcumin induces a release of exosomes depleted in pro-angiogenic proteins and enriched in proteins endowed with anti-angiogenic activity. Among the proteins differential expressed we focused on MARCKS, since it was the most modulated protein and a target of miR-21. Taken together our data indicated that also Curcumin attenuates the exosome's ability to promote the angiogenic phenotype and to modulate the endothelial barrier organization.

## INTRODUCTION

Curcumin is a natural polyphenol derived from the rhizome of the *Curcuma longa* endowed with anti-inflammatory, anti-oxidative and antineoplastic properties. Curcumin is known to inhibit multiple oncogene-driven cell-signaling pathways thus mitigating or preventing many different types of cancers, including colorectal, pancreatic, breast, prostate and lung cancers, in both animal models and humans [1]. It was also demonstrated that Curcumin affects the expression of several miRs, small non coding regulatory RNAs that modulate gene expression by targeting the 3′ untranslated region of mRNA [2].

In leukemic cancer cells, Curcumin induces cell death by apoptosis and inhibits cancer cell proliferation by upregulating the expression of miR-15a and miR-16, resulting in the decreased expression of the antiapoptotic Bcl-2 and in the downregulation of WT1, an oncogene involved in leukemogenesis [3]. Recently, we demonstrated that Curcumin treatment of Chronic myelogenous leukemia (CML) cells caused a selective sorting of miR-21 in exosomes and a concomitant decrease of this miRNA into the cells, thus leading to the upregulation of PTEN and the subsequent inhibition of leukemic cell growth [4].

Angiogenesis is a complex process that depends on the interaction between growth factors, cytokines and a number of components of the extracellular matrix [5, 6]. Sabatel et al. demonstrated that miR-21 over-expression reduced the angiogenic capacity of HUVECs [6] by directly targeting RhoB, a Rho GTPase, 83% identical to RhoA, that is involved in the regulation of cell growth, cellular signaling and cytoskeleton reorganization [7]. In particular, endothelial cell-cell junctions maintain a restrictive barrier that is tightly regulated to allow dynamic responses to permeability-inducing angiogenic factors. These angiogenic stimuli induce a transient remodeling of adherens junctions (AJs), such as VE-Cadherin, depending on Rho GTPase-controlled cytoskeletal rearrangements [8].

In this work we showed that exosomes released by CML cells treated with Curcumin (Curcu-exosomes) contain a large amount of miR-21 which, after its delivery in HUVECs, is able to downregulate RhoB. Thus, the resulting downregulation is able to affect the endothelium monolayer integrity by modulation of tight junctions (ZO-1) and adherent junction (VE-cadherin) proteins. In order to achieve a wider comprehension on the molecular mechanisms mediated by exosomes, underlying the modulation of angiogenesis, we also performed a proteomic profile of exosomes using a SWATH-MS approach. Proteomic analyses of exosomes released by K562 cells treated with Curcumin, compared with exosomes released by control cells, revealed that Curcu-exosomes are depleted in several proteins involved in migration and angiogenesis, while enriched in anti-angiogenic proteins.

Among the proteins which are modulated in Curcu-exosomes in comparison with control exosomes, we focused our attention on MARCKS (myristoylated alanine-rich C-kinase substrate), a phosphoprotein specifically targeted by miR-21 [9] that is a well-established regulator of migration in multiple cell types [10].

Overall, these data suggested that Curcumin treatment reverted the angiogenic effect of exosomes released by CML cells. Curcu-exosomes attenuated the effects of CML exosomes on the endothelium due to changes in their proteomic composition and in the shuttling of miR-21.

## RESULTS

### Curcumin quantification in exosomes

Exosomes released by K562 and LAMA84 cells treated or not with Curcumin (10, 20 and 40 μM) during a 24 hours culture period, were isolated from culture medium and collected [4]. Aliquots of samples were used to quantify Curcumin in exosomes by HPLC analysis.

Table 1 shows the amount of Curcumin extracted from exosomes released by K562 and LAMA84 cells treated with different concentrations (10, 20 and 40 μM) of Curcumin. Values are the mean ± SD of 3 samples. The results indicated that the Curcumin content in exosomes increased as a function of Curcumin concentrations added to both cell lines.

**Table 1:** Curcumin quantification in exosomes

Cell lines	Curcumin treatment	Curcumin in exosome (ng/μg of exosomes)
K562	10 μM	0.035 ± 0.009
	20 μM	0.175 ± 0.031
	40 μM	1.376 ± 0.206
LAMA84	10 μM	0.091 ± 0.018
	20 μM	0.239 ± 0.024
	40 μM	1.083 ± 0.162

### Viability assay of HUVECs treated with Curcu-exosomes

**Figure 1:**
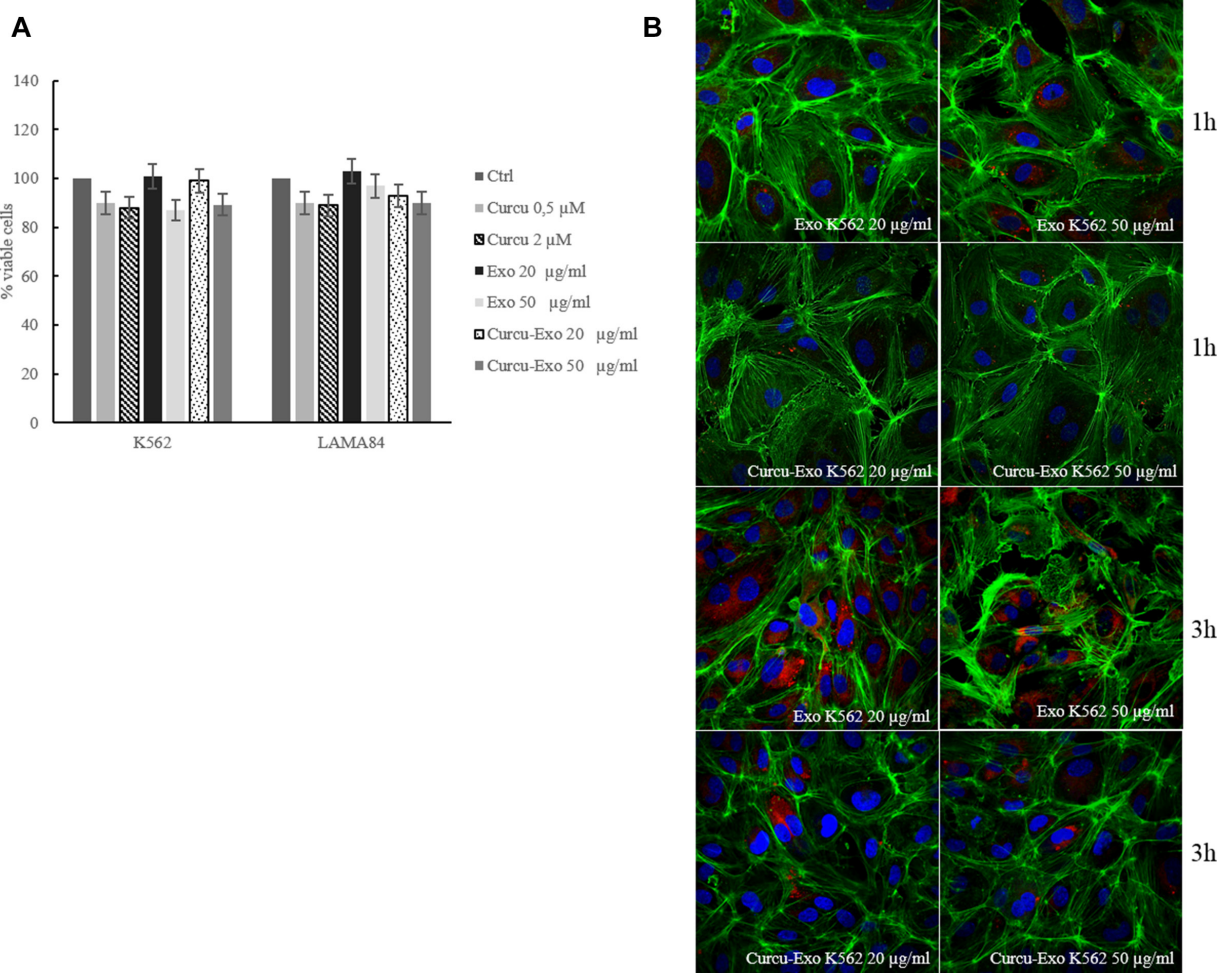
(**A**) HUVECs cell viability was measured by MTT assay after treatment with Curcu-Exo (20–50 μg/ml), Curcumin (0.5 and 2 μM) was used as negative control. The values were plotted as a percentage of viable cells. Each point represents the mean ± SD of three independent experiments. (**B**) Analysis at confocal microscopy of HUVECs treated, for 1 and 3 hours, with 20 μg/ml (Exo 20 μg/ml) and 50 μg/ml (Exo 50 μg/ml) of K562 exosomes, compared with HUVECs treated, for 1 and 3 hours, with 20 μg/ml (Curcu-Exo 20 μg/ml) and 50 μg/ml (Curcu-Exo 50 μg/ml) of exosomes released from K562 cells treated with 20 μM Curcumin. HUVECs were stained with ActinGreen (green), nuclear counterstaining was performed using Hoechst (blue); exosomes were labelled with PKH26 (red).

Exosomes released by CML cells after treatment for 24 hours with 20 μM of Curcumin contain small amounts of Curcumin (Table 1). Curcu-exosomes (20 and 50 μg/ml) were used to treat HUVECs. We decided to use the treatment with 20 μM Curcumin because we also demonstrated that these exosomes were particularly enriched in miR-21 [4]. Cells viability was analyzed using the MTT assay. The results indicated that the treatment with Curcu-exosomes and CML control exosomes, did not affect the endothelial cells (EC) viability (Figure 1A).

### Uptake of Curcu-exosomes by HUVECs

CML cells treated for 24 hours with 20 μM of Curcumin, released vesicles that were isolated, purified on a sucrose gradient and characterized as exosomes, as demonstrated in our previous paper [4]. In order to verify the ability of endothelial cells to uptake K562 Curcu-exosomes, we treated HUVECs with exosomes labeled with PKH-26.

As shown Figure 1B, HUVECs internalized K562 Curcu-exosomes with a slower kinetic than K562 control exosomes. We obtained similar results after treatment of EC with Curcu-exosomes released by LAMA84 cells treated or not with Curcumin (Supplementary Figure S1A). The semi-quantitative analysis of exosomes internalization, measured as red fluorescence intensity in the cytoplasm of HUVECs is shown in Supplementary File, Supplementary Figure S1, S1B and S1C.

### Curcu-exosomes shuttled miR-21 into HUVECs

**Figure 2:**
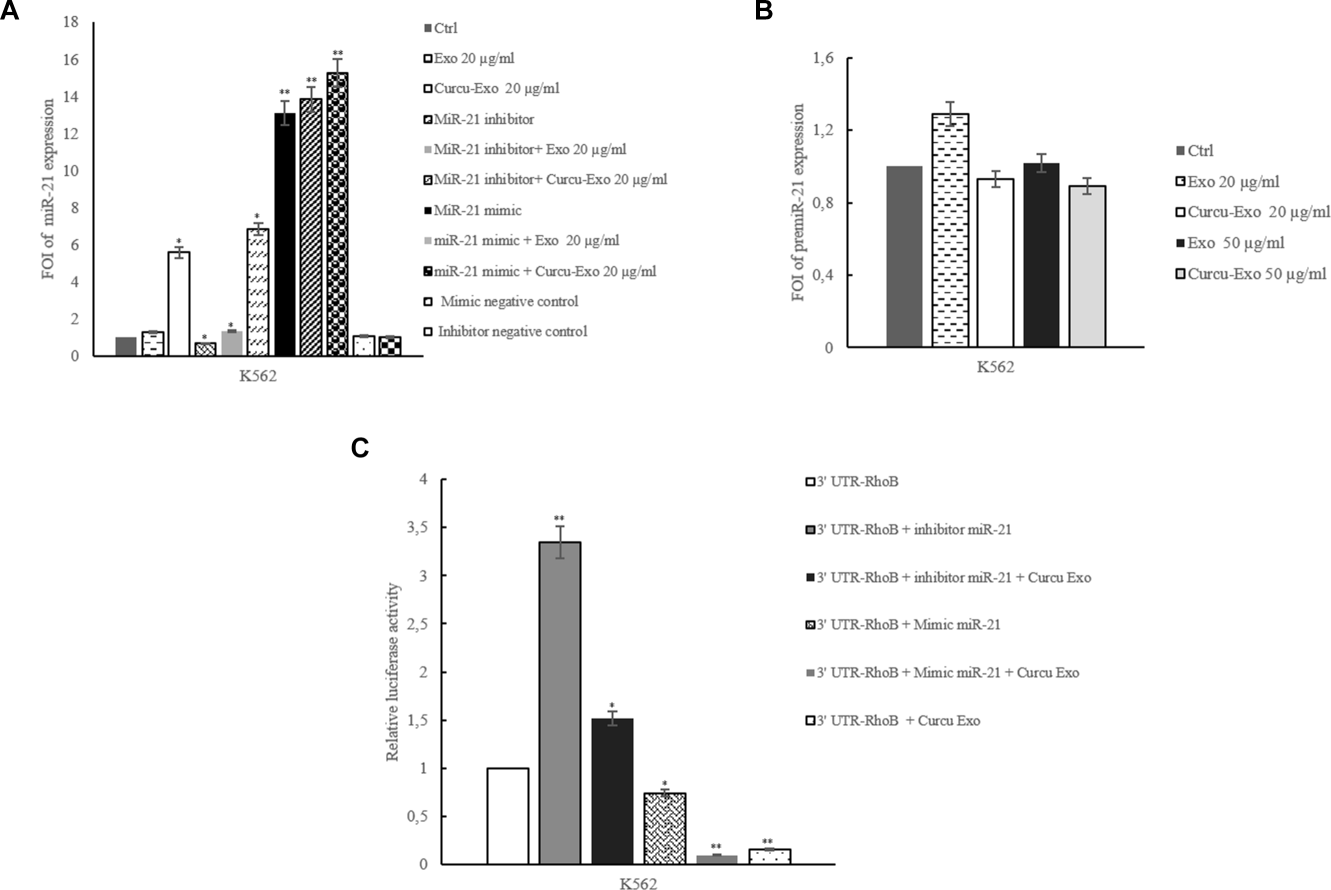
MiR-21 expression in HUVECs treated with exosomes released by K562 treated or not with Curcumin (**A**) miR-21 expression levels in HUVECs treated with 20 μg/ml of K562 Curcu-exosomes and control exosomes were determined by quantitative Real time PCR analysis. We also analyzed miR-21 expression in HUVECs treated with K562 Curcu-exosomes and control exosomes and/or transfected with miR-21 inhibitor or miR-21 mimic. Values (FOI: fold of induction) are the mean ± SD of 3 independent experiments *p ≤ 0.05, **p ≤ 0.01. (**B**) Pre-miR-21 expression in HUVECs treated with different amounts of K562 exosomes. Pre-miR-126 expression levels in HUVECs treated with 20 and 50 μg/ml of K562 exosomes were determined by quantitative Real time PCR analysis. We also analyzed pre-miR-21 expression in HUVECs treated with K562 Curcu-exosomes and control exosomes and/or transfected with miR-21 inhibitor or miR-21 mimic. (**C**) Luciferase activity of HUVECs transfected with reporter plasmid (RhoB-pEZX), treated with K562 Curcu-exosomes and control exosomes and/or cotransfected with miR-21 inhibitor or miR-21 mimic.

We recently demonstrated that the treatment of CML cells with Curcumin causes an increase of miR-21 in released exosomes [4]. In order to demonstrate the transfer of miR-21 in HUVECs, we treated endothelial cells with 20 and 50 μg/ml of Curcu-exosomes released by K562 cells and we analyzed the expression levels of miR-21 in HUVECs. As shown in Figure 2A, miR-21 levels increased in HUVECs treated with Curcu-exosomes compared with untreated or control exosomes-treated HUVECs.

In HUVECs transfected with miR-21 inhibitor (2′-OMe-miR-21), we observed a decrease of miR-21 expression but the treatment with Curcu-exosomes reverted this effect.

On the contrary, we observed that the increased level of miR-21 in HUVECs transfected with miR-21 mimic was further augmented after addition of Curcu-exosomes (Figure 2A). In order to exclude the possibility that Curcumin-treated CML exosomes could induce the expression of endogenous miR-21 in HUVECs, we quantified the levels of precursor miR-21 (pre-miR-21) in HUVECs by Real Time PCR. As shown in Figure 2B, we found no statistically significant difference of pre-miR-21 expression level after treatment of EC with 20 and 50 μg/ml of K562 Curcu-exosomes. We obtained similar results after the treatment of endothelial cells with Curcu-exosomes released by LAMA84 cells treated or not with Curcumin (Supplementary Figure S2A and S2B).

### MiR-21 targets RhoB 3′-UTR mRNA

MiRNA target prediction algorithm indicates that RhoB is a predictive target of miR-21. MiR-21 directly targets Rho B and miR-21 overexpression is known to affect endothelial cell migration and organization into capillary like structures [6].

We confirmed that miR-21 binds to RhoB 3′UTR mRNA using a Firefly/Renilla Duo-Luciferase reporter vector (pEZX-MT01) where the 3′ UTR of RhoB was cloned downstream of the firefly luciferase gene (RhoB-pEZX). When HUVECs, transfected with reporter plasmid, were incubated with 20 μg/ml of Curcu-exosomes, the firefly luciferase activity was decreased as compared with untreated cells or HUVECs treated with exosomes control (Figure 2C). Down regulation of miR-21, by transfection of miR-21 inhibitor into HUVECs, containing RhoB-pEZX, increased the activity of firefly luciferase with respect to untransfected HUVECs. The treatment of HUVECs with K562 Curcu-exosomes transfected with RhoB–pEZX and miR-21 inhibitor reverted the effect of miR-21 inhibitor. In contrast, luciferase activity decreased when HUVECs containing RhoB-pEZX were transfected with miR-21 mimic, similarly to treatment with Curcu-exosomes. The treatment of HUVECs, transfected with RhoB–pEZX and miR-21 mimic, with Curcu-exosomes released by K562 cells, reinforced the effect of miR-21 mimic (Figure 2C). We obtained similar effects in HUVECs, transfected with miR-21 mimic and inhibitor, treated with exosomes released by LAMA84 cells treated with Curcumin (Supplementary Figure S2C).

### Treatment of HUVECs with Curcu-exosomes inhibited RhoB expression

**Figure 3:**
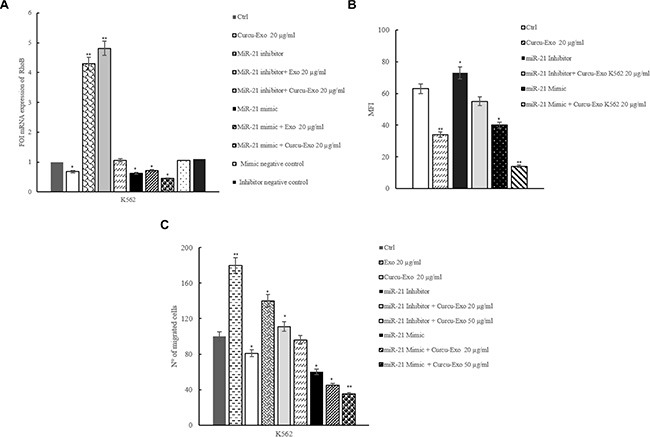
MiR-21, shuttled by exosomes, modulates RhoB expression in HUVECs (**A**) Real time PCR analysis showed that RhoB mRNA expression decreased in HUVECs treated with Curcu-exosomes compared to control exosomes. Expression of RhoB was evaluated in HUVECs transfected with 2-Ome-miR-21 (miR-21 inhibitor) treated or not with 20 μg/ml of control exosomes (miR-21 inhibitor + Exo 20 μg/ml) and Curcu-exosomes (miR-21 inhibitor + Curcu-Exo 20 μg/ml). Expression of RhoB was also evaluated in HUVECs transfected with miR-21 mimic (miR-21 mimic) treated or not with 20 μg/ml of control exosomes (miR-21 mimic + Exo 20 μg/ml) and Curcu-exosomes (miR-21 mimic + Curcu-Exo 20 μg/ml). Values (FOI: fold of induction) are the mean ± SD of 3 independent experiments *p ≤ 0.05, **p ≤ 0.01. (**B**) Histogram shows the MFI (Mean Fluorescence Intensity) relative to the expression of RhoB in HUVECs after treatment with low serum medium (Control), 20 μg/ml of exosomes (Exo 20 μg/ml) and 20 μg/ml of Curcu-exosomes (Curcu-exo 20 μg/ml). Expression of RhoB was evaluated, with FACS analysis, in HUVECs transfected with 2-Ome-miR-21(miR-21 inhibitor) and treated with 20 μg/ml of control exosomes (miR-21 inhibitor + Exo 20 μg/ml) and Curcu-exosomes (miR-21 inhibitor + Curcu-Exo 20 μg/ml). Surface expression of VCAM1 was evaluated in HUVECs transfected with miR-21 mimic (miR-21 mimic) and treated with 20 μg/ml of control exosomes (miR-21 mimic + Exo 20 μg/ml) and 20 μg/ml of Curcu-exosomes (miR-21 mimic + Curcu-Exo 20 μg/ml). Values are the mean ± SD of 3 independent experiments *p ≤ 0.05 **p ≤ 0.01. (**C**) Curcu-exosomes inhibit HUVECs migration. Addition of control exosomes (20, 50 μg/ml) to the upper wells of the chamber induces dose-dependent increase of HUVEC migration, the addition of Curcu-exosomes reverts this effects. Values are the mean ± SD of 3 fields in three independent experiments *p ≤ 0.05, **p ≤ 0.01. The ability of migrating of HUVECs transfected with 2-Ome-miR-21 (miR-21 inhibitor) treated or not with 20 μg/ml of control exosomes (miR-21 inhibitor + Exo 20 μg/ml) and with 20 μg/ml of Curcu-exosomes (miR-21 inhibitor + Curcu-Exo 20 μg/ml), was evaluated. The ability of migration of HUVECs transfected with miR-21 mimic (miR-21 mimic) treated or not with 20 μg/ml of control exosomes (miR-21 mimic + Exo 20 μg/ml) and with 20 μg/ml of Curcu-exosomes (miR-21 mimic + Curcu-Exo 20 μg/ml) was also measured.

Our results showed that miR-21 targets the 3′ UTR of RhoB mRNA. By real time PCR analysis, we evaluated if exosomes treatment of HUVECs induced a modulation of RhoB mRNA expression. As shown in Figure 3A, K562 Curcu-exosomes caused a decrease of RhoB mRNA expression. In HUVECs transfected with miR-21 inhibitor (2′-O Me miR-21), RhoB mRNA expression showed a 3 fold increase compared with untransfected cells. The treatment with Curcu-exosomes of HUVECs transfected with miR-21 inhibitor counteracted the effect of miR-21 inhibitor, decreasing the expression of RhoB mRNA (Figure 3A). Forced expression of miR-21 in HUVECs decreased the relative amount of RhoB mRNA of about 60% (Figure 3A). The addition of Curcu-exosomes, in HUVECs transfected with miR-21 mimic, reinforced the downregulation of RhoB expression (Figure 3A). We obtained similar effects in HUVECs, transfected with miR-21 mimic and inhibitor and treated with exosomes released by LAMA84 cells treated with Curcumin (Supplementary Figure S2D).

Similarly, FACS analyses revealed a decrease of RhoB protein after treatment with Curcu-exosomes. This effect was reinforced when HUVECs were transfected with miR-21 mimic. When HUVECs were transfected with miR-21 inhibitor, the Curcu-exosomes treatment reverted the effect on RhoB decreased expression (Figure 3B).

Our data indicated that Curcu-exosomes treatment induces an exosome-mediated increase of miR-21 that in turn causes the modulation of RhoB expression in endothelial cells, confirmed by the study of gain and loss of function for miR-21 (Figure 3B).

### Curcu-exosomes inhibited the migration of endothelial cells

Endothelial cell migration is a crucial process in angiogenesis. In order to understand the biological effects of RhoB inhibition, due to miR-21 shuttled in exosomes, we analyzed the effect of Curcu-exosomes addition (20 and 50 μg/ml) on HUVECs motility. Figure 3C showed that the treatment with CML exosomes increased the motility of EC, as previously described [4].

Curcu-exosomes treatment inhibited in a dose-dependent manner, the motility of HUVECs towards complete medium. We observed similar effects after the transfection of miR-21 mimic in HUVECs both treated with K562 (Figure 3C) and LAMA84 (Supplementary Figure S3A) exosomes. Conversely, the transfection of miR-21 inhibitor in EC induces an increase in cell motility. The effects observed with the transfection with miR-21 mimic are even more significant after the treatment with Curcu-exosomes (Figure 3C), because of the stronger decrease of HUVECs motility with respect to the effects Curcu-exosomes alone. On the other hand, in endothelial cells transfected with miR-21 inhibitor we observed an increase of HUVECs motility, similar to the treatment with control exosomes. The effects of the transfection with miR-21 inhibitor were reverted after the treatment with Curcu-exosomes (Figure 3C).

### Treatment of HUVECs with Curcu-exosomes modulated IL8 expression and secretion

**Figure 4:**
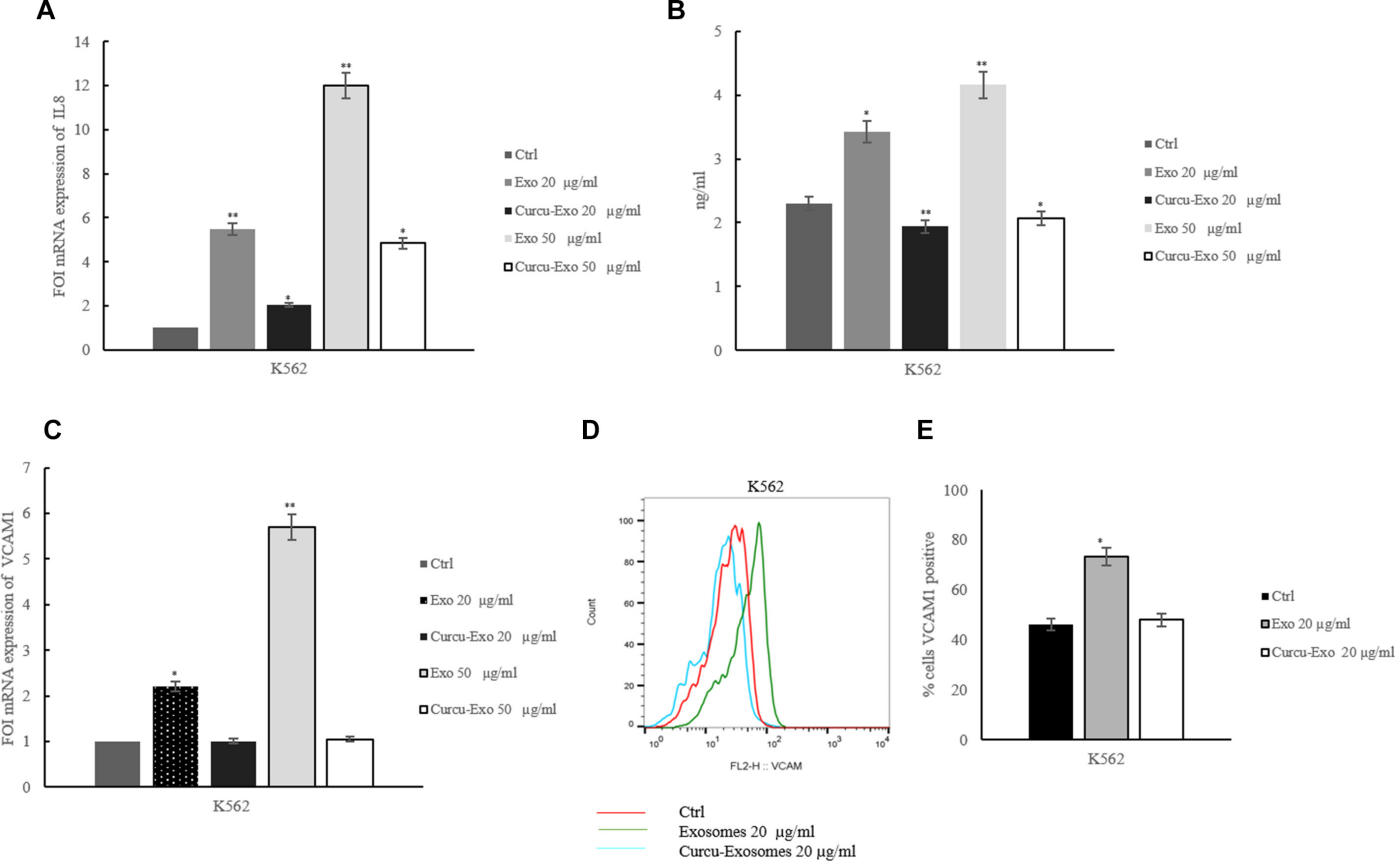
Treatment of HUVECs with Curcu-exosomes modulated IL8 and VCAM1 expression (**A**) Real time PCR analysis showed that IL8 mRNA expression decreased in dose dependent manner in EC treated Curcu-exosomes compared to control exosomes. Values (FOI: fold of induction) are the mean ± SD of 3 independent experiments *p ≤ 0.05, **p ≤ 0.01. (**B**) ELISA assay showed that IL8 protein expression decreased in EC treated with Curcu-exosomes respect to control exosomes, in a dose dependent manner. (**C**) Real time PCR analysis showed that VCAM1 mRNA expression decreased in dose dependent manner in HUVECs treated with Curcu-exosomes compared to control exosomes. Values (FOI: fold of induction) are the mean ± SD of 3 independent experiments *p ≤ 0.05, **p ≤ 0.01. (**D**) FACS analysis showed that VCAM1 protein expression decreased in EC treated with Curcu-exosomes respect to control exosomes. e: Histogram shows the percentage of VCAM1 positive HUVECs after 6 hours of treatment with low serum medium (Ctrl), 20 μg/ml of exosomes (Exo 20 μg/ml) and 20 μg/ml of Curcu-exosomes (Curcu-exo 20 μg/ml). Values are the mean ± SD of 3 fields in three independent experiments *p ≤ 0.05, **p ≤ 0.01.

Tumor angiogenesis is caused by the aberrant production of angiogenic factors secreted by malignant tumor cells, host cells, or both [11]. IL-8 has been shown to regulate pathological angiogenesis, as well as tumor growth and metastasis. As previously demonstrated, CML exosomes added to HUVECs increased IL-8 mRNA level and this effect was reverted after treatment of EC with Curcu-exosomes (Figure 4A). These data were confirmed at protein level by an ELISA assay on conditioned medium of HUVECs treated with 20 and 50 μg/ml Curcu-exosomes and control exosomes. As shown in Figure 4B, the addition of exosomes caused a dose-dependent increase of IL8 released from HUVECs; this effect was reverted after treatment with Curcu-exosomes. We obtained similar results in HUVECs treated with Curcu-exosomes released by LAMA84 cells, both at mRNA (Supplementary Figure S3B) and protein level (Supplementary Figure S3C).

### Treatment of HUVECs with Curcu-exosomes modulated VCAM1 expression

K562 and LAMA84exosomes induced VCAM1 expression after treatment of endothelial cells for 6 hours [12]. As shown in Figure 4C, the addition of increasing doses of exosomes released by CML cells to HUVECs caused a dose-dependent increase in VCAM1 mRNA expression. In endothelial cells, the treatment with Curcu-exosomes reverted this effect. By FACS analysis, we observed an increase of VCAM1 protein in HUVECs treated with control exosomes released by K562 cells. The treatment with Curcu-exosomes reverted this effect and the expression of VCAM1 is comparable to the untreated cells (Figure 4D). Figure 4E showed the percentage of VCAM1 positive EC after treatment with Curcu-exosomes and control. We obtained similar results in HUVECs treated with Curcu-exosomes released by LAMA84 cells, both at mRNA (Supplementary Figure S4A) and protein level (Supplementary Figure S4B and S4C).

### Curcu-exosomes inhibited *in vitro* and *in vivo* tube formation

**Figure 5:**
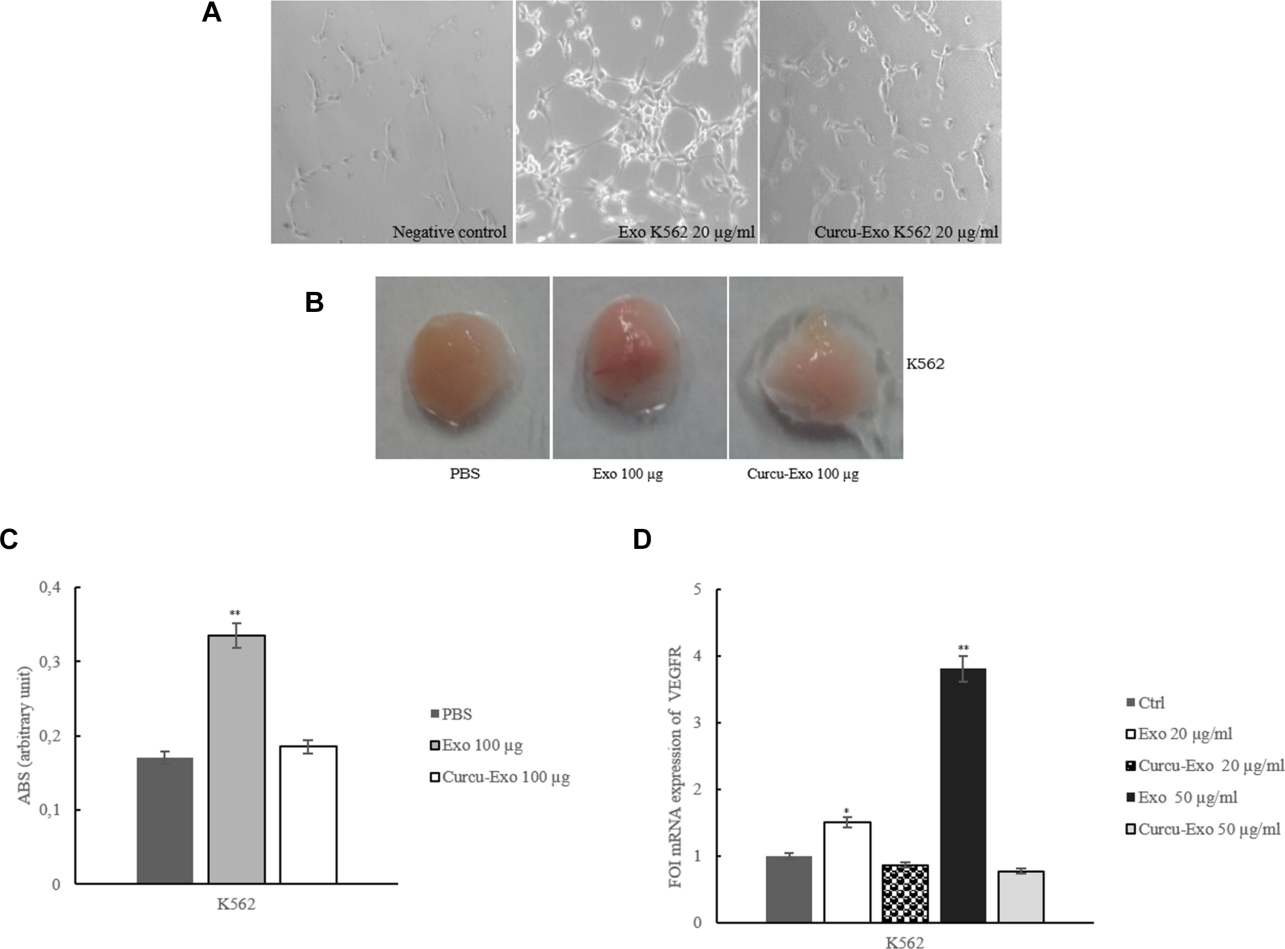
K562 exosomes stimulate *in vitro* and *in vivo* angiogenesis (**A**) Phase contrast micrographs showing that K562 control exosomes induce an endothelial network formation on Matrigel, K562 Curcu-exosomes revert this effect. No tube formation is observed when HUVECs are plated in low-serum medium (negative control). (**B**) Matrigel plug containing K562 exosomes stimulate angiogenesis in mice (Exo 100 μg), this effect revert when K562 Curcu-exosomes (Curcu-Exo 100 μg) are used. PBS was used as a negative control. (**C**) Haemoglobin concentration in the exosomes-containing Matrigel was evaluated with Dabkin's assay. (**D**) Real time PCR analysis showed that VEGFR mRNA expression decreased in EC treated with Curcu-exosomes compared to control exosomes. Values (FOI: fold of induction) are the mean ± SD of 3 independent experiments *p ≤ 0.05, **p ≤ 0.01.

We analyzed the ability of HUVECs to form capillary-like structures when plated on Matrigel as an *in vitro* model of angiogenesis. HUVECs maintained in low serum medium were unable to form a tube network (Figure 5A). Addition of CML exosomes induced, as described, an endothelial network formation [12], while the treatment with Curcu-exosomes inhibited the development of capillary structures (Figure 5A, Curcu-Exo).

The angiogenic potential of CML exosomes was then assayed *in vivo* by examining the recruitment of vasculature into subcutaneously implanted Matrigel plugs containing exosomes [12, 13]. Plugs containing control CML exosomes became more vascularized in comparison to the implants with Curcu-exosomes (Figure 5B). This observation is supported by the decreased haemoglobin concentration in the Curcu-exosomes-containing Matrigel plugs with respect to control exosomes-containing Matrigel plugs (Figure 5C). We obtained similar results with Curcu-exosomes released by LAMA84 cells (Supplementary Figure S4D, S4E and S4F).

Interestingly, HUVECs cells treated with control K562 exosomes showed an increase in VEGFR mRNA expression levels compared to the treatment with Curcu-exosomes from K562 (Figure 5D) and LAMA84 cells (Supplementary Figure S4G).

### Effect of Curcu-exosomes on endothelial cell tight and adherent junction

**Figure 6:**
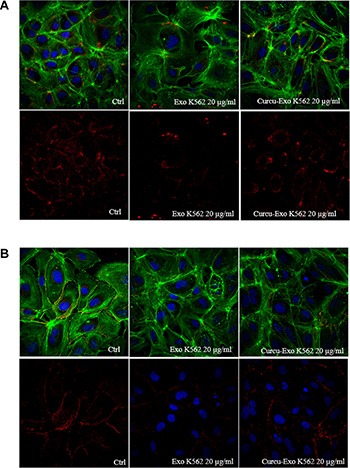
Alteration of HUVEC monolayer after addition of K562 exosomes (**A**) Analysis at confocal microscopy of ZO-1 localization in HUVECs treated with K562 exosomes (Exo K562 20 μg/ml) revealed a decrease of immunostaining compared to untreated cells (Ctrl). The treatment with K562 Curcu-exosomes (Curcu-Exo K562, 20 μg/ml) reverted this effect. (**B**) Analysis at confocal microscopy of VE Cadherin localization in HUVECs treated with K562 exosomes (Exo K562 20 μg/ml) revealed a decrease of immunostaining compared to untreated cells (Ctrl). The treatment with K562 Curcu-exosomes reverted this effect (Curcu-Exo K562 20 μg/ml).

In order to elucidate the effects of Curcu-exosomes on endothelial barrier stabilization, in particular on tight junctions, we analyzed by immunofluorescence, the localization of ZO1 in control EC and Curcu-exosomes treated cells. In HUVECs, the addition of 20 μg/ml of control exosomes caused a delocalization of ZO1 compared to HUVECs control in which ZO1 was localized in plasma membrane. In HUVECs treated with 20 μg/ml of Curcu-exosomes released by K562 (Figure 6A) and LAMA84 (Supplementary Figure S5A) cells, a localization of ZO1 in HUVECs plasma membrane similar to control cells was observed.

We also determined the effects of exosomes on the expression of VE-Cadherin, an endothelial specific transmembrane adhesion molecule. Control cells had a continuous peripheral VE-Cadherin staining; on the contrary, the staining intensity decreased in HUVECs after treatment with 20 μg/ml of exosomes released by K562 (Figure 6B) and LAMA84 (Supplementary Figure S5C) control cells. Interestingly this destabilizing effect was reverted after treatment with Curcu-exosomes, thus supporting our hypothesis that Curcu-exosomes modulate endothelial barrier stabilization.

### Permeability of HUVEC monolayers

**Figure 7:**
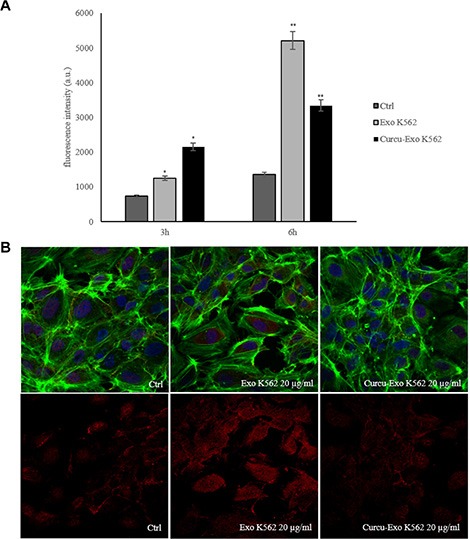
Vascular permeability is modulated by K562 control and Curcu-exosomes (**A**) The permeability of HUVEC monolayer increased after treatment, for 3 and 6 hours, with K562 control exosomes (Exo K562) compared to untreated HUVEC monolayer (Ctrl), the treatment with Curcu-exosomes (Curcu-Exo K562), protected the endothelial monolayer. (**B**) Upper panel: Analysis at confocal microscopy of endothelial monolayer. The integrity of the monolayer was altered, after treatment with K562 control exosomes (Exo K562 20 μg/ml); the treatment of Curcu-exosomes alleviated the alteration of the EC monolayer (Exo K562 20 μg/ml). Lower panel: Analysis at confocal microscopy of RhoB expression in HUVECs treated with K562 Curcu-exosomes and control exosomes. K562 control exosomes (Exo K562 20 μg/ml) induce an increase of immunostaining for RhoB compared to untreated cells (Ctrl). The treatment with K562 Curcu-exosomes (Curcu-Exo K562, 20 μg/ml) reverted this effect.

Vascular permeability is a parameter of endothelial cell function and its alterations are a feature of different processes, including cancer metastasis. In order to investigate if Curcu-exosomes alleviated the alteration of HUVEC monolayers we performed a permeability assay with FITC-dextran. As showed in Figure 7A, upper panel, the permeability of HUVEC monolayer increased after treatment, for 3 and 6 hours, with CML exosomes, while the treatment with Curcu-exosomes partially protected the endothelial monolayer. Quantitative analysis of fluorescence revealed a five fold increase of permeability after the addition of control exosomes. That increase was then reverted after treatment with Curcu-exosomes.

The confocal analyses confirmed that Curcu-exosomes protected the integrity of HUVECs monolayer with respect to control exosomes. As shown in Figure 7B, after treatment with CML exosomes the integrity of the monolayer was altered. Conversely, the treatment of endothelial cells with Curcu-exosomes, alleviated the alteration of the EC monolayer, but caused a rearrangement of actin, in concordance with RhoB inhibition (Figure 3A). As shown in Figure 7B, lower panel, the treatment with K562 Curcu-exosomes (Curcu-Exo K562 20 μg/ml) of the endothelial monolayer caused a decrease of RhoB expression compared to the treatment with K562 control exosomes (Exo K562 20 μg/ml). These observations suggested that Curcu-exosomes protected the EC monolayer and reduced the vascular permeability.

### Proteomic analyses of exosomes released by K562 cells treated or not with Curcumin

Tumor cell-derived exosomes can modulate target cells phenotype through the deliver of both RNAs and cargo proteins. To better investigate how exosome proteins from curcumin-treated K562 cells can mediate the anti-angiogenic effect observed on HUVECs, a proteomic analysis using a SWATH-MS approach was performed (for Materials and Methods see online Supplementary Information), on exosomes released by K562 cells treated or not with Curcumin. SWATH analysis is a quantitative label-free method that combines the high specificity of Data Independent Acquisition (DIA) method with a targeted data extraction strategy. Only proteins with a *p* ≤ 0.05 and a log10 fold-change > 0.2 or < −0.2 (for upregulated and downregulated proteins respectively) were considered for further analysis. A total of 30 proteins, differentially regulated in exosomes released by K562 cells following treatment with curcumin, were identified; in comparison with exosomes secreted by K562 control cells, Curcu-exosomes showed 4 up-regulated and 26 down-regulated proteins (Supplementary Tables S1 and S2). A GO enrichment analysis on all differentially expressed proteins was performed using iPathway Guide (http://www.advaitabio.com/ipathwayguide.html) highlighting a significant enrichment for those GO terms linked with biosynthetic processes and translation. In particular, the following GO terms resulted significantly enriched: intracellular transport (*p*-value = 0.008; BP), translation (*p*-value = 0.009; BP), cellular biosynthetic process (*p*-value = 0.0018; BP), nucleic acid binding (*p*-value = 0.016; MF), anion transmembrane transporter activity (*p*-value = 0.016; MF), nitric-oxide synthase regulator activity (*p*-value = 0.028; MF), structural constituent of ribosome (*p*-value = 0.033; MF), ribonucleoprotein complex (*p*-value = 0.013; CC), nucleus (*p*-value = 0.014; CC), ribosomal subunit (*p*-value = 0.015; CC.). Among them, nitric-oxide synthase regulator activity, referred to hsp90aa1 and hsp90ab1 proteins, is particularly interesting, since the nitric oxide (NO) pathway appears to play a key role in tumor angiogenesis and spread. Heat shock protein 90 (HSP90) binds directly to endothelial NO synthase (eNOS), promoting its activity and augmenting NO production [14]. Surprisingly, an accurate MEDLINE search allowed us to discover that approximately 50% of modulated protein dataset was involved with the angiogenic, invasive and metastatic processes (Table 2). Among these proteins, we focused our attention on MARCKS-related protein (MARCKSL1) because it is the mainly down-regulated protein, with a 10.6-fold down-regulation. Interestingly, miR-21 has been shown to specifically target the mRNA of MARCKS. So, we decided to further investigate the role played by this protein in the anti-angiogenic effect of curcumin.

**Table 2:** Angiogenesis- and migration-related proteins

AC^a^	Protein name (Gene name)	Curcu-Exo vs Control Exo^b^		Description	References
		*Fold Change*	*p-Value*		
P49006	MARCKS-related protein (MARCKSL1)	−10.6	0.000590	Regulates of actin assembly dynamics during migration in multiple types of cells. Regulation of migration in multiple cell types; its dysregulation is associated with metastasis and is an indicator of poor prognosis. Increase of cell motility in many cell types (fibroblasts, glial cells, macrophages, neutrophils, skeletal myoblasts, endothelial cells and VSMCs) through several molecular mechanisms: protein kinase B pathway, direct binding to actin, control of PIP2 availability at the plasma membrane and regulation of the small GTPases, Rac1 and Cdc42. Key mediator of the H_2_O_2_-induced permeability change in bovine aortic endothelial cells.	[15–18]
P13164	Interferon-induced transmembrane protein 1 (IFITM1)	−7.2	0.000022	Promotion of cancer progression by enhancing cell migration and invasion in gastric cancer and head and neck cancer. Its knockdown inhibits migration and invasion by decreasing expression and activity of MMP9, in glioma cells. Key Role in the formation of functional blood vessels; stabilization of EC-EC interactions during endothelial lumen formation by regulating tight junction assembly.	[19–22]
O43707	Alpha-actinin-4 (ACTN4)	−3.2	0.004910	Enhancement of cancer cell motility, invasion, and metastasis; concentration at the leading edge of migrating cells.	[23]
H0YDJ9	Tetraspanin (CD81)	−3.1	0.012240	Components of the endothelial lateral junctions implicated in the regulation of cell motility. Involvment in cell migration of tumor and immune cells. Its association with the small GTPase Rac limits the GTPase activation within the plasma membrane so contributing to regulation of Rac activity turnover.	[24, 25]
J3KPF3	4F2 cell-surface antigen heavy chain (SLC3A2)	−2.9	0.032730	Regulation of tumor cell migration, proliferation, spreading and survival *in vitro*. Transport of L-arginine, required for NO synthesis in HUVECs, in association with SLC7A6 or SLC7A7.	[26, 27]
P35613	Basigin (BSG)	−2.6	0.000042	Regulation of expression of vascular endothelial growth factor (VEGF) and MMPs in stromal cells; stimulation of the production of MMPs in human umbilical vein endothelial cells (HUVECs) and of VEGF expression in tumor compartment. Involvement in angiogenesis through different ways such as the modulation of VEGF isoforms secretion.	[28–34]
P63244	Guanine nucleotide-binding protein subunit beta-2-like 1 (GNB2L1)	−2.5	0.006900	Massive up-regulation in vascular endothelial cells during angiogenesis *in vitro* and *in vivo* and in human carcinoma cells. Involvement in Gbg-mediated adherens junction assembly in endothelial cells. Regulation of VEGF-Flt1-dependent cell migration of endothelial cells and macrophages through direct interaction with Flt1 and activation of PI3K/Ak-Rac1.	[35–39]
P02792	Ferritin light chain (FTL)	−2.1	0.020850	Binding to a 22-aa subdomain of Hka, so antagonizing antiangiogenic effects of this last and enhancing the migration, assembly and survival of HKa-treated endothelial cells.	[40]
P08238	Heat shock protein HSP 90-beta (HSP90AB1)	−1.7	0.036260	Exposure of endothelial cells to VEGF triggers the association of HSP90 with VEGFR2, that drives the phosphorylation of FAK on Tyr407 in a RhoA-ROCK-dependent manner, and recruitment of paxillin and vinculin to FAK so leading to the assembly of focal adhesions and endothelial cell migration.	[41]
O00299	Chloride intracellular channel protein 1 (CLIC1)	−1.6	0.006680	Strong expression in endotheliall cells; important in multiple steps of *in vitro* angiogenesis and in regulation of cell surface expression of various integrins acting in angiogenesis. Mediation of endothelial cell growth, branching morphogenesis and migration, possibly via regulation of integrin expression.	[42]
P08567	Pleckstrin (PLEK)	4.2	0.044860	Its overexpression in COS-1 cells leads to a reorganization of the actin cytoskeleton partially dependent on Rac1 but independent of PI3K and Cdc42. Stabilization of apical junctional adhesion complexes (AJCs), that were composed of adherens junctions and tight junctions and are involved in cell-to-cell adhesion, by bridging transmembrane cadherins to the intracellular microtubule network of proteins.	[43, 44]
E9PPJ5	Midkine (MDK)	3.5	0.000280	Abrogation of the VEGF-A–induced proliferation of human microvascular endothelial cells *in vitro* through the downregulation of proangiogenic cytokines and through the upregulation of antiangiogenic factors. Downregulation of VEGF-A–induced neovascularization and vascular permeability *in vivo*.	[45]

### Treatment of HUVECs with Curcu-exosomes modulated MARCKS expression

**Figure 8:**
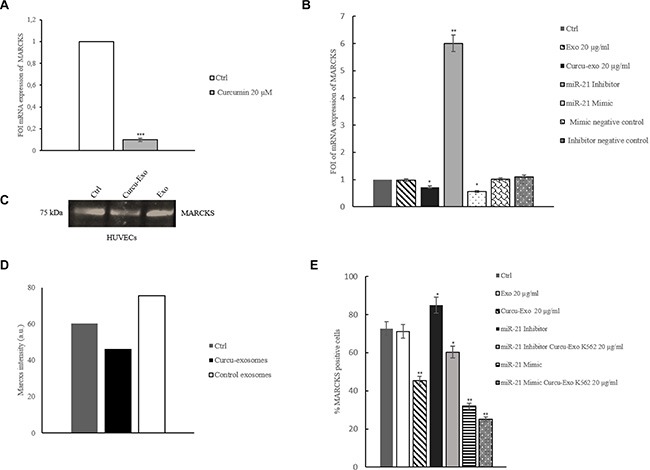
Curcumin modulates MARCKS expression in K562 cells and HUVECs (**A**) Real time PCR analysis showed that in K562 cells MARCKS mRNA expression decreases after treatment with Curcumin respect to untreated cells. Real time PCR analysis showed that MARCKS mRNA expression decreased in HUVECs treated with Curcu-exosomes respect to control exosomes. (**B**) Expression of MARCKS was evaluated in HUVECs transfected with 2-Ome-miR-21 (miR-21 inhibitor) treated or not with 20 μg/ml of control (miR-21 inhibitor + Exo 20 μg/ml) and Curcu-exosomes (miR-21 inhibitor + Curcu-Exo 20 μg/ml). Expression of MARCKS was also evaluated in HUVECs transfected with miR-21 mimic (miR-21 mimic) treated or not with 20 μg/ml of control exosomes (miR-21 mimic + Exo 20 μg/ml) and Curcu-exosomes (miR-21 mimic + Curcu-Exo 20 μg/ml). Values (FOI: fold of induction) are the mean ± SD of 3 independent experiments *p ≤ 0.05, **p ≤ 0.01. (**C**) Western blotting analyses of MARCKS in HUVECs treated with Curcu-exosomes and control exosomes. (**D**) Densitometric analysis of Western blotting against MARCKS. (**E**) Facs analysis of MARCKS expression.

Recently, miR-21 has been shown to directly target MARCKS, binding in the 3′ untranslated region from the nt713–734 of 3′ UTR mRNA. MARCKS are known to affect the architecture of the actin cytoskeleton in endothelial cells, modulating EC motility and permeability [10]. In order to confirm the data which have been obtained with proteomic analyses, we evaluated MARCKS expression at mRNA and protein level. By Real time PCR analysis, we evaluated MARCKS expression in K562 cells treated with Curcumin. As showed in Figure 8A, Curcumin induced a decrease of MARKCS mRNA expression. Moreover, Curcu-Exosomes treatment of HUVECs induced a modulation of MARCKS mRNA expression. In HUVECs transfected with miR-21 inhibitor (2′-O Me miR-21), MARCKS mRNA expression showed a 5 fold increase with respect to untransfected cells. Forced expression of miR-21 in HUVECs caused a 60% decrease of the relative amount of MARCKS mRNA (Figure 8B).

At protein level, we observed an increase of MARCKS in HUVECs treated with control exosomes compared to untreated HUVECs, while the treatment with Curcu-exosomes reverted this effect (Figure 8C). We also observed by FACS analyses a decrease of MARCKS after treatment with Curcu-exosomes, and this effect was reinforced when HUVECs transfected with miR-21 mimic were treated with Curcu-exosomes. In these, we further observed a low increase of MARCKS expression when compared with untrasfected cells. The treatment of HUVECs transfected with miR-21 inhibitor with Curcu-exosomes has proved to be able to revert the effect on MARCKS decreased expression (Figure 8E). These data confirmed that Curcu-exosomes transport miR-21, biologically active, that inhibits MARCKS.

## DISCUSSION

Exosomes play a critical role in mediating cell-to-cell communication. Several studies have demonstrated that exosomes modulate angiogenic process [5, 12, 46]. Our research group discovered that CML cells affect vascular remodeling in *in vitro* and *in vivo* models through the IL8 modulation in endothelial cells. We also demonstrated that exosomes released from CML cells stimulate bone marrow stromal cells to produce IL8 that, in turn, is able to affect both *in vitro* and *in vivo* leukemia cell malignant phenotype [47].

Recently, the critical role played by miRNA delivered by exosomes in the typical cell-to-endothelial cell communication in leukemia has been investigated. In our previous paper, we demonstrated that miR-126 shuttled by CML exosomes is biologically active in targeting endothelial cells and affects CML cell motility and adhesion [46]. Umezu et al. observed that exosomes, collected from miR-92a-overexpressing leukemia cells, entered into endothelial cells, resulting in an enhanced migration and tube formation [48]. These data indicated that exosomal miRNAs have an important role in tumor-endothelial crosstalk occurring in the bone marrow microenvironment, potentially affecting disease progression.

Furthermore, it was demonstrated that some natural compounds can alter gene expression involved in cancer progression [2]. Curcumin, a natural compound present in turmeric, has been recognized as a promising anti-cancer drug and is being developed as a chemopreventive agent in various cancers. Preclinical studies have shown that Curcumin has antioxidant, anti-inflammatory and antiproliferative activities. Curcumin affects several molecules involved in biochemical and molecular cascades, via direct molecular interactions and epigenetic modulation of gene expression. The modulation of miRNA expression by Curcumin has been shown in several models [49]. The effects of Curcumin on miRNAs expression was initially studied in pancreatic cancer cells [50]. Curcumin was reported to downregulate the expression of 18 miRNAs including miR199a* known to target MET proto-oncogene and the downstream extracellular signal-regulated kinase 2 [51]. It was also described that Curcumin, in retinoblastoma cells, upregulates the expression of 11 miRNAs including miR-22 that leads to a decreased expression of its downstream genes including the transcription factor SP1, implicated in the growth and metastasis of several cancer types [52].

We demonstrated that Curcumin caused a decrease of cellular miR-21 in CML cells while increased miR-21 selective packaging in exosomes. Furthermore, we showed that addition of Curcumin to CML cells caused a downregulation of Bcr-Abl expression through the cellular increase of miR-196b. Curcumin, therefore, alters miRs expression that may contribute to its antileukemic effect in CML [4]. On the other hand, miRs have been reported to play an important role in several functions of endothelial cells, including migration [6] and angiogenesis [53]. It was demonstrated that miR-21 overexpression affects endothelial cell migration and organization into capillary-like structures. MiR-21 was also found to modify actin cytoskeleton organization, thus affecting cell migration and angiogenesis [6].

In this study, we showed that exosomes released by CML cells after Curcumin treatment deeply changed their molecular composition, acquiring antiangiogenic properties. Curcu-exosomes were enriched in miR-21 [4], which was then shuttled in endothelial cells (Figure 2A) as a biologically active form.

Our data are in agreement with the results of Sabatel et al., demonstrating that miR-21 over-expression reduces the angiogenic capacity of HUVECs [6], targeting directly RhoB, a critical regulator of actin dynamics. RhoB is a Rho GTPase whose expression is inducible by a variety of stimuli including growth factors. Most Rho GTPases act on membranes and affect the movement of cell membranes by changing the membrane-associated cytoskeletal actin. Endothelial Rho proteins are also involved in ICAM1 mediated signaling events [54]. Rho may be activated through cell-surface signals propagated to the cytoskeletal actin [55].

Curcu-exosomes inhibit the expression of RhoB at protein and mRNA level (Figure 3A and 3B), via miR-21 transport, thus resulting unable to promote the angiogenic phenotype in endothelial cells. The biological effects of Curcu-exosomes on endothelial barrier stabilization were confirmed with a permeability assay (Figure 8A).

Several studies have also shown that Curcumin is able to counteract the stimuli-induced production of IL8 through the modulation of NF–κB, JNK, ERK1/2, and p38 pathways. Wang et al. showed that Curcumin had an inhibitory effect on the expression of IL8 induced by DEHP [56]. We observed that CML exosomes added to HUVECs were able to increase IL8 at mRNA and protein level. We also proved that this effect was reverted after treatment of EC with Curcu-exosomes (Figure 5A and 5B). The antiangiogenic effect of Curcu-exosomes was reinforced by decreasing the expression of VCAM1 at mRNA and protein level with respect to control exosomes. (Figure 5C, 5D and 5E). These antiangiogenic effects were confirmed with *in vitro* and *in vivo* angiogenic assays.

Kalani and colleagues showed that exosomes derived from Curcumin-treated endothelial cells alleviated oxidative stress, affecting tight junctions (ZO-1, claudin-5, occludin) and adherent junction (VE-cadherin) proteins and mitigated the endothelial cell layer permeability induced during EC damage due to high homocysteine levels [57]. It was also demonstrated that Curcumin had a beneficial effect on blood brain barrier (BBB), under ischemic conditions, protecting the tight junction from a possible dysfunction and ameliorating the BBB permeability [58]. We also observed that Curcu-exosomes have protective effects on endothelial barrier, specifically it stabilizes tight and adherent junctions. As it has been showed with an immunofluorescence assay, in HUVECs treated with control exosomes, we observed a delocalization of ZO1 and VE-Cadherin with respect to control HUVECs in which ZO1 and VE-Cadherin was localized in plasma membrane. This effect reverted in HUVECs treated with CML Curcu-exosomes.

Interestingly our SWATH analysis of exosomes released by control and Curcumin treated cells evidenced a relevant and significant modulation of several proteins involved in the angiogenic process. In particular, proteomic data highlighted that Curcumin induced the release of exosomes depleted in pro-angiogenic proteins such as: Interferon-induced transmembrane protein 1 (IFITM1), actinin 4, basigin, Guanine nucleotide-binding protein subunit beta-2-like 1 (GNB2L1) and MARCKS [59], and enriched in proteins, such as Pleckstrin [43] and Midkine [45], which present known antiangiogenic abilities (Table 2). We focused our attention on MARCKS, since it was the most modulated protein and also a target of miR-21. MARCKS is a phosphoprotein that belongs to the myristoylated alanine-rich C-kinase substrate (MARCKS) family and possesses actin-binding properties through which it is implicated in control of cell motility [59]; MARCKS dysregulation is closely associated with metastasis in a wide range of cancers and it is also an indicator of poor prognosis.

MARCKS expression in Curcu-exosomes was about 11 fold less than control exosomes; moreover in HUVECs treated with Curcu-exosomes, enriched in miR-21, we observed a decrease of MARCKS (Figure 5). The downregulation of this protein was shown to contribute to maintain the vascular integrity by stabilizing the endothelial junctions.

Overall, these observations demonstrated that Curcumin induced a modification of CML exosomes composition at protein and microRNA level. Curcu-exosomes were able to modulate the endothelial barrier organization and attenuated the angiogenic phenotype. Our data suggest that Curcumin could be a potential therapeutic agent for CML treatment with a double effect, on cancer cells and on tumor microenvironment.

## MATERIALS AND METHODS

### Cell culture and reagents

HUVECs were obtained from Lonza (Clonetics, Verviers, Belgium) and grown in endothelial growth medium (EGM) according to supplier's information. K562 and LAMA84, chronic myelogenous leukemia cells were cultured as previously described [60]. All other reagents were purchased from Sigma (St. Louis, MO), if not otherwise cited.

### Curcumin quantification in exosomes by HPLC assay

HPLC analyses were performed with a Shimadzu LC-10AD_VP_ instrument equipped with a binary pump LC-10AD_VP_, a UV SPD-M20A Diode Array detector, a 20 μL injector and a computer integrating apparatus (EZ Start 7.3 software). HPLC solvents (HiPerSolvChromanorm) were purchased from VWR International (Milan, Italy). Chromatographic separation was achieved on a reversed-phase column Chromolith^®^ Performance (RP-18e, Merck, 100 × 4.6 mm) and a mobile phase consisted of methanol [Mobile phase A] and TFA 0.01% (v/v) aqueous solution [Mobile phase B]. The developed gradient method consisted of % change in mobile phase B with respect to time (0.01→3.00 min: 99.5% B; 3.01→15.00 min: 12% B; 15.01→16.99 min 12% B; 17.00→25.00 min 0% B; 25.01→30.00 min 99.5% B). The mobile phase was filtered through Whatman filter 0.45 μm and degassed before use. The flow rate was set at 1 mL/min and the UV wavelength at 257, 280, 400 and 420 nm. In these conditions, the retention time for Curcumin was 17.1 minutes. Preparation of stock and working standard solutions was performed as follow: 50 mg of curcumin working standard dissolved in 50 mL of acetone. This stock standard solution was diluted to prepare six sets of calibration standards of curcumin at concentrations range of 0.02–1.00 μg/mL in acetone. (LOQ = 0.01 μg/ml). The standard calibration curve was constructed using peak area versus known concentrations of curcumin. HPLC reports were highly reproducible and linearly related to concentration (regression equation y = 54144 ×– 84.22; R 0.9944; R^2^ 0.9888). The linear regression line was used to determine the linearity and concentration of the samples.

200 μl of exosome samples (2 μg/μl in protein content determined by Bradford method), isolated by K562 and LAMA84 cells treated or not with Curcumin (10, 20 and 40 μM), were added to 200 μl of methanol and centrifuged at 14.000 rpm for 10 min; after that, the supernatant (20 μl) was analyzed by HPLC as described above. Each sample was analyzed in triplicate.

### Proliferation assay (MTT assay)

Methyl-thiazol-tetrazolium (MTT) assay was executed as previously described [11]. HUVECs were plated in triplicate at 5 × 10^4^ per well and treated with Curcu-exosomes (20 and 50 μg/ml) for 24 hours. Means and standard deviations generated from three independent experiments are reported as the percentage of viable cells.

### Exosomes isolation

Exosomes released by K562 and LAMA84 cells treated or not with Curcumin (10, 20 and 40 μM) during a 24 hours culture period were isolated from the culture medium supplemented with 10% FBS (previously ultracentrifuged) by different centrifugations, as previously described [12, 61], and isolated vesicles were purified on a 30% sucrose/D2O cushion. Vesicles contained in the sucrose cushion were recovered, washed, ultracentrifuged for 90 min in PBS and finally collected for further uses. Exosome's protein content was then determined with the Bradford method.

### qPCR for RhoB, VCAM1, IL8, MARCKS, VEGFR, ZO1 and VE-Cadherin

Total cellular RNA was isolated from HUVECs and incubated for 6 hours with exosomes released by K562 and LAMA84 cells treated or not with curcumin, using the RNAspin Mini (GE Healthcare Science, Uppsala, Sweden). For RhoB, IL8, VCAM1, ZO1, VE-Cadherin, VEGFR and MARCKS mRNA detection, 1 μg of total RNA was reverse transcribed using the High Capacity cDNA Archive kit (Life Technologies, Carlsbad, California, U.S.), according to manufacturer's instructions. RT-QPCR was performed in 48-well plates using the Step-One Real-Time PCR system (Applied Biosystem). For quantitative SYBER^®^Green Real Time PCR, reactions were carried out in a total volume of 20 μl containing 2× SYBR^®^Green I Master Mix (Applied Biosystems), 2 μl cDNA and 300 nM forward and reverse primers. Primers sequence were: GAPDH (5′ATGGGGAAGGTGAAGGTCG3′, 5′GGGTCATTGAT GGCAACAATAT3′), MARCKS (5′TGCTCTTTGCCA CCCGATAA3′, 5′ACCCTAAAACTGCACACTGCT3′), VEGFR (5′CGGTCAACAAAGTCGGGAGA3′, 5′CAGT GGCACCACAAAGACACG3′) ZO1 (5′ TTAAGCCAG CCTCTCAACAGAAA3′, 5′GGTTGATGATGCTGGGT TTGT3′), and VE-Cadherin (5′GATCAAGTCAAGCGTG AGTCG3′; 5′AGCCTCTCAATGGCGAACAC3′). RhoB, IL8, VCAM1 transcript levels were measured by TaqMan Real Time PCR, using the TaqMan gene expression assay for IL8 (HS00174103 m1), VCAM1 (Hs00174239 m1), RhoB (Hs03676562 m1) and GAPDH (Hs99999905 m1), all obtained from Invitrogen (Foster City, CA, USA).

Real-time PCR was performed in duplicate for each data point. Relative changes in gene expression between control and treated samples were determined with the ΔΔCt method. Changes in the target mRNA content relative to GAPDH were determined using the comparative Ct method as described in the previous paragraph.

### Quantitative polymerase chain reaction (qPCR) for miRNAs and pre-miRNAs

The expression of miR-21 was tested by miScript PCR System (QIAGEN, Hilden, Germany). Total cellular RNA and miRNAs were isolated from HUVECs incubated or not with exosomes using the RNAspin Mini (GE Healthcare Science, Uppsala, Sweden). Reverse transcription reactions were performed using miScript II RT Kit (QIAGEN, Hilden, Germany) as described by the manufacturer's instructions. We used miScriptHiSpec Buffer for cDNA synthesis to detect mature miRNA and miScriptHiFlex Buffer for cDNA synthesis to enable the quantification of precursor miRNA. Quantitative Real Time PCR was performed using miScript SYBR Green PCR Kit (QIAGEN, Hilden, Germany). Mature miR-21 was detected by miScript Primer Assay and precursor miR-21 by miScript Precursor Assays according to manufacturer's instructions. RNU6-2 was used as endogenous control. Expression levels of miRNAs and pre-miRNA were determined using the comparative Ct method to evaluate changes in Ct and ultimately fold and percent change. An average Ct value for each RNA was obtained from triplicate reactions.

### Transfection of HUVECs with miR-21 mimic or inhibitor

Transfection of miScript miR-21 inhibitor (QIAGEN, Hilden, Germany) or miScript miR-21 mimic in CML cells (QIAGEN, Hilden, Germany) was performed according Fast-Forward Transfection protocol (QIAGEN, Hilden, Germany). 6 × 10^4^ K562 and LAMA84 cells per well were seeded in a 24-well plate in 500 μl of RPMI. MiScript miR-21 (2′-O-Me-miR-21) or miScript miR-21 mimic (2 μM) were diluted in 100 μl of culture medium without serum to obtain a final 5 nM miRNA concentration. The cells were transfected using HiPerFect Transfection Reagent (QIAGEN, Hilden, Germany) for 18 h according to manufacturer's instructions. MiScript Inhibitor Negative Control (Inhibitor Negative Control) (QIAGEN, Hilden, Germany) and AllStars Negative Control siRNA (Mimic Negative Control) (QIAGEN, Hilden, Germany) were used as negative controls as indicated by manufacture's technical specifications. Transfection efficiency was evaluated by quantitative Real Time PCR.

### Luciferase activity assay

The 3′-UTRs of RhoB was cloned in pEZX-MT01 vector (Genecopoeia, Rockville, MD USA). The construct's designs were based on the sequence of miR-21 binding sites. 8 × 10^4^ HUVECs per well in a 24 well plate were seeded in 500 μl of an appropriate culture medium, the cells were transfected with 300 ng of the pEZX-MT01 firefly luciferase report diluted in 60 μl culture medium without serum. HUVECs were cotransfected with 6 pmol of miR-21 mimic or miR-21 inhibitor using Attractene Transfection Reagent (QIAGEN, Hilden, Germany) according to manufacturer's protocol.

To test if exosomal miR-21 targets RhoB mRNA, HUVECs were incubated with 20 μg/ml of exosomes released by CML cells treated or not with Curcumin, after the transfection with pEZXMT01vector. Firefly and Renilla Luciferase activities were measured consecutively using the kit Dual Glo^®^ Luciferase Assay System (Promega Corp., Madison, WI, USA) 24 hours after transfection using GloMax^®^-Multi Detection System (Promega Corp., Madison, WI, USA). Each transfection was repeated twice in duplicate.

### ELISA for IL8

Conditioned medium (CM) of HUVECs, transfected or not with miR-21 mimic or inhibitor, and treated with 20 and 50 μg/ml of Curcu-exosomes and control exosomes, was collected from the cells after 6 hours of incubation. CM aliquots were then centrifuged to remove cellular debris and IL8 protein levels were measured using an ELISA kit (Invitrogen Carlsbad, California, USA), according to the manufacturer's protocol.

### Motility assay

HUVECs treated with 20 and 50 μg/ml of Curcu-exosomes were suspended in serum-free RPMI 1640 medium supplemented with 0.1% BSA in transwells with 8 μm pore filters and exposed to complete RPMI 1640 as chemoattractant for 6 hours. After incubation, cells migrated in the bottom wells were counted.

### Permeability assay

The transendothelial permeability of HUVECs monolayers was measured using Transwell polycarbonate insert filters (Corning, Little Chalfont, UK pore size 0.4 μm). HUVECs were grown as a tight monolayer and treated for 3 and 6 hours with Curcu-exosomes and exosomes released by control cells. FITC-dextran (500 kDa, Sigma) was added at EC monolayers in 200 μl of culture medium for 30′. Samples were removed from the lower chamber for fluorescence measurements. Fluorescence was measured using Glomax.

### HUVEC tube formation on Matrigel

Matrigel was used to test the effects of exosomes on the *in vitro* vascular tube formation as previously described [12]. Exosomes released by K562 and LAMA84 cells treated or not with Curcumin (20 μM), were added to HUVECs plated on Matrigel in endothelial basal medium containing 0.2% of FBS as indicated. Cells were incubated for 3 hours and then evaluated by phase-contrast microscopy and photographed.

### Matrigel plug assay

All animal experiments were conducted in full compliance with University of Palermo and Italian Legislation for Animal Care. Four weeks old BALB/c mice (Charles River Laboratories International, Wilmington, MA) were injected subcutaneously with 200 μl Matrigel (BD Biosciences Pharmingen, San Diego, CA) containing 100 μg K562 and LAMA84 cells-derived exosomes treated or not with Curcumin or PBS (negative control). After 4 weeks the plugs were collected and the degree of vascularization was evaluated by determination of hemoglobin content using the Drabkin method (Drabkin's reagent kit) [12].

### Immunofluorescence analysis

HUVEC monolayers were grown to confluence on coverslips coated with type I collagen (Calbiochem, Darmstadt, Germany) and were treated with 20 μg/ml of CML Curcu-exosomes, control exosomes or low serum medium for 6 hours. After incubation, cells were processed as previously described [7]. Antibodies used in the experiments were anti-RhoB (1:100; Novus), anti-ZO1 (1:100; Santa Cruz technology) and anti VE-Cadherin (1:100; Santa Cruz technology). Cells were stained with Texas Red-conjugated secondary anti mouse antibodies (1:100; Molecular Probe, Eugene, OR) and analysed by confocal microscopy (Nikon A1).

### Uptake of LAMA84 and K562 exosomes by HUVECs

K562 and LAMA84 exosomes were labeled with PKH26 according to supplier's information. Briefly, exosomes collected after the 100,000 × g ultracentrifugation, were incubated with PKH26 for 10 min at room temperature. Labeled exosomes were washed in PBS by ultracentrifugation; the pellets were resuspended in low serum medium and incubated with HUVECs for 1 and 3 hours. HUVECs were grown on coverslips coated with type I collagen (Calbiochem, Darmstadt, Germany) and were treated with increasing doses of K562 and LAMA84 Curcu-exosomes or control exosomes. HUVECs were stained with Actin Green (Molecular Probes, Life Technologies, Carlsbad, California, U.S) that binds actin with high affinity. Nuclei were stained with Hoechst (Molecular Probes, Life Technologies, Carlsbad, California, U.S) and analysed by confocal microscopy. Each picture was acquired with adjustments to the laser intensities and to the amplifier gains to avoid pixel saturation. Each fluorophore used was excited independently and sequential detection was performed. One single picture consisted of a z-series of images of 1024–1024 pixel resolution. The semi-quantitative analysis of fluorescence intensity was performed using IMAGE-J software (http://imagej.nih.gov/ij/).

### FACS analyses

Expression of cell surface VCAM1 in HUVECs was determined by flow cytometry analysis. HUVECs were incubated for 6 h with 20 μg/ml of K562 and LAMA84 exosomes in a low serum medium (EGM: RPMI, 1:9). 2 × 10^5^ cells were washed in PBS and incubated with anti-VCAM1-PE antibody (20 μl) (BD Bioscences, Mountain View, CA, USA) for 15 min at 4°C according to manufacturer's recommendations. Isotype-matched irrelevant antibodies were used as a negative control. Viable cells were gated by forward and side scatter and analysis was performed on 100,000 acquired events for each sample. Samples were analysed on a FACS Calibur with the use of the CellQuest software (BD Biosciences, NJ, USA).

Expression of HUVECs intracellular RhoB was determined by flow cytometry analysis. HUVECs, transfected or not with miR-21 mimic and inhibitor, were treated with 20 μg/ml of K562 exosomes treated or not with curcumin for 6 h. After treatment, the cells were centrifuged for 5 min at 300 × g and washed with PBS/BSA. 100 μl of Fixation buffer (reagent A of Leucoperm, AbDSerotec) was added for 15 min at room temperature, then the cells were washed in PBS/BSA and centrifuged for 5 min at 300 × g. Cells were resuspended with 100 μl of permeabilization buffer (reagent B of Leucoperm, AbDSerotec). Soon after, a RhoB unconjugated primary antibody (Novus Biologicals) was added for 30 min at room temperature. Cells were washed with PBS/BSA and a FITC secondary antibody was added for 15 min. Stained cells were washed with PBS/BSA and analysed on a FACS Calibur (Becton Dickinson) using Cellquest software.

### Proteomic analyses: Sample preparation, SWATH-MS and data analysis

250 μg of exosomes released by curcumin-treated and control K562 cells were subjected to in-solution digestion using 50% 2,2,2-trifluoroethanol (TFE) in PBS, as previously described (Principe et al.), with some modifications. Briefly, after 2 minutes sonication in an ice bath, exosomes were incubated with constant shaking for 2 h at 60°C; extracted exosomal proteins were reduced with 5 mM DTT for 30′ at 60°C, alkylated with 25 mM iodoacetamide (IAA) for 30′ in the dark at room temperature and digested for 18 hours adding trypsin at a ratio of 1:50 (w/w). After stopping digestion, extracted peptides were desalted using C18 Macrospin columns, dried and resuspended in 5% acetonitrile (ACN)/H2O (5:95, v/v) containing 0.1% formic acid (FA). A pool containing an equal amount of all three samples has been prepared to generate the spectral reference library for SWATH-MS analysis.

All the analyses were performed using a Triple TOF 5600 Plus System (AB Sciex, Framingham, U.S.A.) equipped with an Eksigent ekspert nano LC 425 system. After being cleaned and pre-concentrated on a C18 reverse-phase trap column, employing a mobile phase, from loading pump, containing 0.1% v/v FA in water at a flow rate of 5 μl/min, peptides were separated on the C18 analytical column, equilibrated at 40°C with a solvent A (0,1% FA in water), at a flow rate of 300 nL/min, using a 100 min gradient method: (10–40% solvent B over 60 min, 40–70% solvent B over 15 min; 70–95% solvent B over 1 min, hold solvent B at 95% for 5 min, 95–10% solvent B in 1 min and hold solvent B at 10% for remaining 18 min. The solvent B was 98% ACN and 0,1% FA. To generate the spectral reference library, the pooled sample was subjected to traditional Information Dependent Acquisition (IDA). The mass spectrometer was operated such that an MS scan (400–1250 m/z; accumulation time 250 ms) analyzed TOF in high resolution mode (> 30,000), followed by 50 MS/MS scans (230–1500 m/z, accumulation time 65 ms) analyzing TOF in high sensitivity mode (resolution > 15,000) with rolling collision energy. For fragmentation, precursors, with charges from 2 to 5, were selected if exceeding a threshold of 100 counts per second (cps); former ions were excluded for 12s. The IDA file was submitted to Protein Pilot™ 4.5 software (AB SCIEX, Toronto, Canada) using Uniprot as human protein database. The search was performed with the following settings: identification as sample type, iodoacetamide cysteine alkylation, digestion by trypsin; no special factors; run of false discovery rate analysis; protein pilot score > 0.05 with a 10% confidence threshold.

For SWATH acquisition, peptides were analysed in SWATH-MS mode. At a cycle time of 2s, 50 ms TOF/MS survey scan was performed between 400–1250Da with 34 × 25 Da swath. Each SWATH MS/MS acquisition was performed between 230–1500 Da using a 76 ms accumulation time. Data from three independent experiments were acquired for each sample. The SWATH files were processed by Peak View v2.2 and Marker View. In Peak View they were analyzed using the following parameters: 10 peptides; 7 transitions; peptide confidence threshold of 90%; FDR threshold of 5%; exclusion of modified peptides; XIC Extraction Window of 15 min; XIC width set at 75 ppm. Protein list with FDR lower than 5% was exported to Marker View for statistical data analysis. Gene ontology and pathway analyses were performed using iPathway Guide (http://www.advaitabio.com/ipathwayguide).

## SUPPLEMENTARY MATERIAL FIGURES AND TABLES




