# Supplementary material for: Curcumin modulates chronic myelogenous leukemia exosomes composition and affects angiogenic phenotype via exosomal miR-21
Source: Oncotarget. 2016 Mar 30;7(21):30420–39. doi: 10.18632/oncotarget.8483 (PMC5058690; doi:10.18632/oncotarget.8483)
Supplement: Supplementary file 1 [file oncotarget-07-30420-s001.pdf]

Curcumin modulates chronic myelogenous leukemia exosomes composition and affects angiogenic phenotype *via* exosomal miR-21

Supplementary Materials

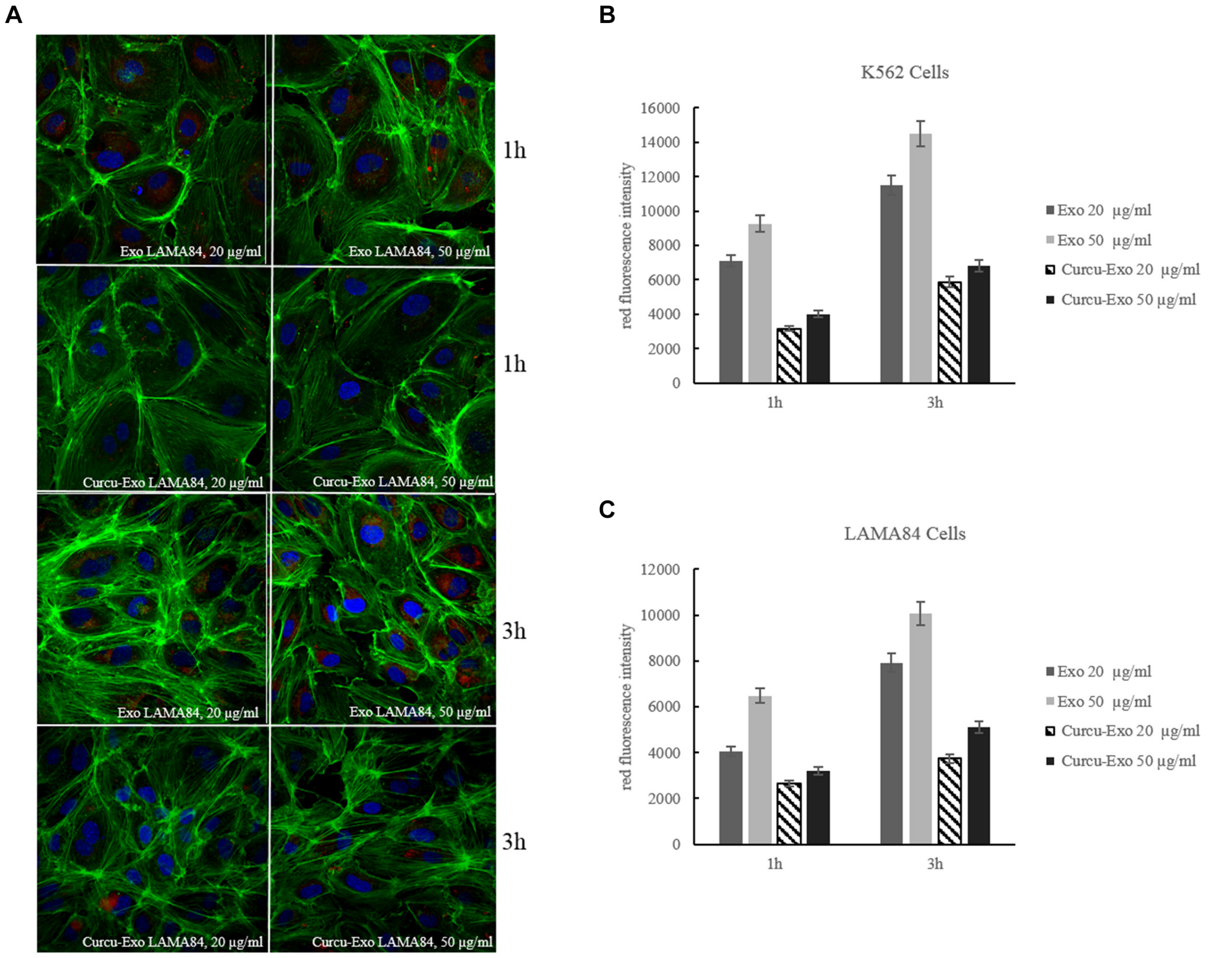

Supplementary Figure S1: (**A**) Analysis at confocal microscopy of HUVECs treated, for 1 and 3 hours, with 20 µg/ml (Exo 20 µg/ ml) and 50 µg/ml (Exo 50 µg/ml) of LAMA84 exosomes, compared with HUVECs treated, for 3 hours, with 20 µg/ml (Curcu-Exo 20 µg/ ml) and 50 µg/ml (Curcu-Exo 50 µg/ml) of exosomes released from LAMA84 treated with 20 µM Curcumin. HUVECs were stained with ActinGreen (green), nuclear counterstaining was performed using Hoescht (blue); exosomes were labelled with PKH26 (red). (**B**) The semi-quantitative analysis of K562 exosomes internalization, measured as red fluorescence intensity in the cytoplasm of HUVECs. (**C**) The semi-quantitative analysis of LAMA84 exosomes internalization, measured as red fluorescence intensity in the cytoplasm of HUVECs.

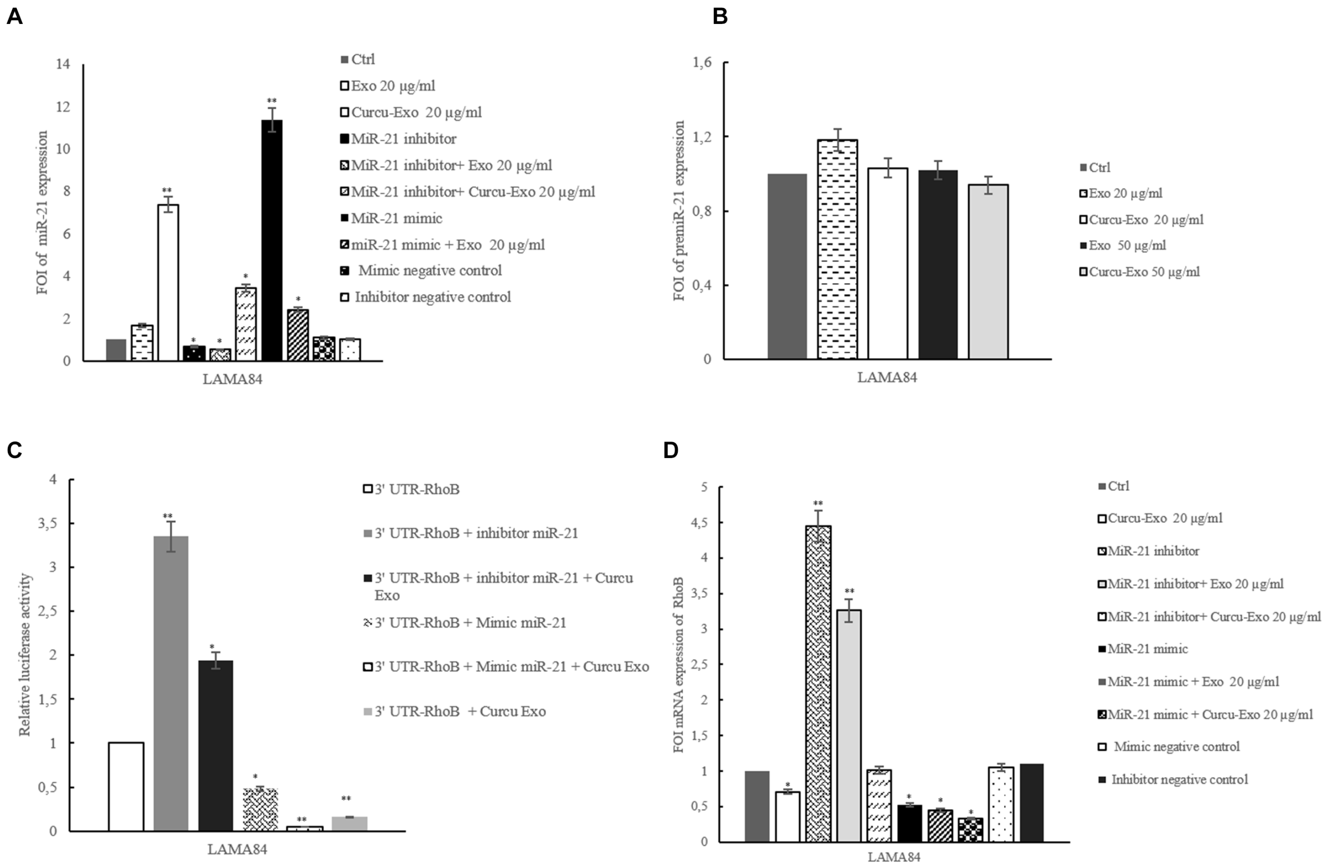

Supplementary Figure S2: MiR-21 expression in HUVECs treated with exosomes released from LAMA84 treated or not with Curcumin. (**A**) miR-21 expression levels in HUVECs treated with 20 µg/ml of LAMA84 Curcu-exosomes and control exosomes were determined by quantitative Real time PCR analysis. Values are the mean ± SD of 3 independent experiments $*p \leq 0.05$, $**p \leq 0.01$. (**B**) Pre-miR-21 expression in HUVECs treated with different amounts of LAMA84 exosomes. Pre-miR-126 expression levels in HUVECs treated with 20 and 50 µg/ml of LAMA84 exosomes were determined by quantitative Real time PCR analysis. (**C**) Luciferase activity of HUVECs transfected with reporter plasmid (RhoB-pEZX), treated with LAMA84Curcu-exosomes and control exosomes and/or cotransfected with miR-21 inhibitor or miR-21 mimic. (**D**) Real time PCR analysis showed that RhoB mRNA expression decreased in LAMA84 cells treated with Curcu-exosomes compared to control exosomes. Expression of RhoB was also evaluated in HUVECs transfected with miR-21 inhibitor (2-Ome-miR-21) treated or not with 20 µg/ml of LAMA84 control and Curcu-exosomes (2-Ome-miR-21 + Exo 20 µg/ml) and in HUVECs transfected with miR-21 mimic (miR-21 mimic) treated or not with 20 µg/ml of exosomes (miR-21 mimic + Exo 20 µg/ml). Values are the mean ± SD of 3 independent experiments $*p \leq 0.05$, $**p \leq 0.01$.

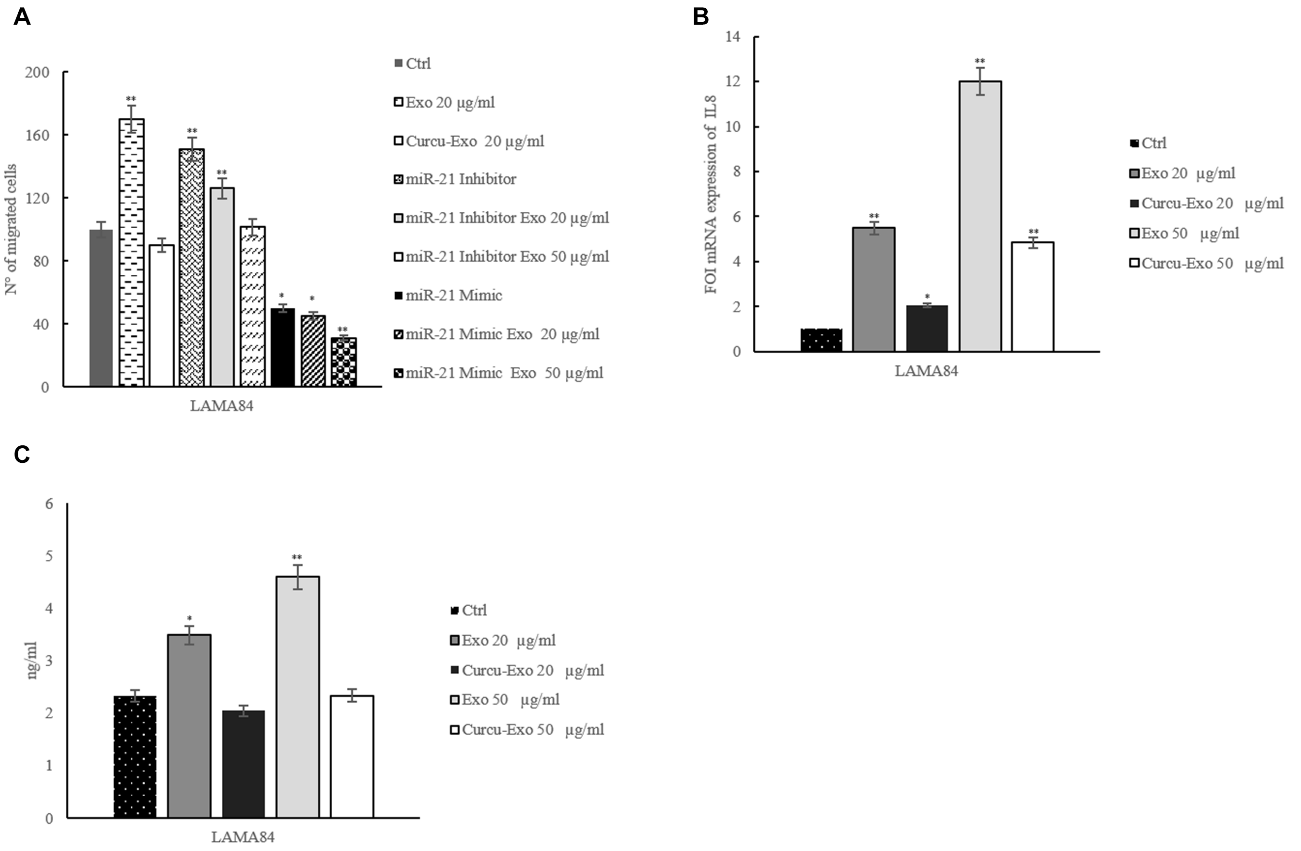

Supplementary Figure S3: Curcu-exosomes inhibit HUVECs migration. (**A**) Addition of control exosomes (20, 50 µg/ml) to the upper wells of the chamber induces a dose-dependent increase of HUVEC migration, the addition of Curcu-exosomes reverts these effects. Values are the mean ± SD of 3 fields in three independent experiments $*p \leq 0.05$, $**p \leq 0.01$. The migration ability of HUVECs transfected with miR-21 inhibitor (2-Ome-miR-21) and treated or not with 20 µg/ml of LAMA84 control and Curcu-exosomes (2-Ome-miR-21 + Exo 20 µg/ml) and in HUVECs transfected with miR-21 mimic (miR-21 mimic) and treated or not with 20 µg/ml of exosomes (miR-21 mimic + Exo 20 µg/ml) was also evaluated. (**B**) Treatment of HUVECs with Curcu-Exosomes modulated IL8 expression. Real time PCR analysis showed that IL8 mRNA expression in dose dependent manner in HUVECs after addition of Curcu-exosomes compared to control exosomes. (**C**) ELISA assay showed that IL8 protein expression decreased in EC treated with Curcu-exosomes compared to control exosomes, in dose dependent manner.

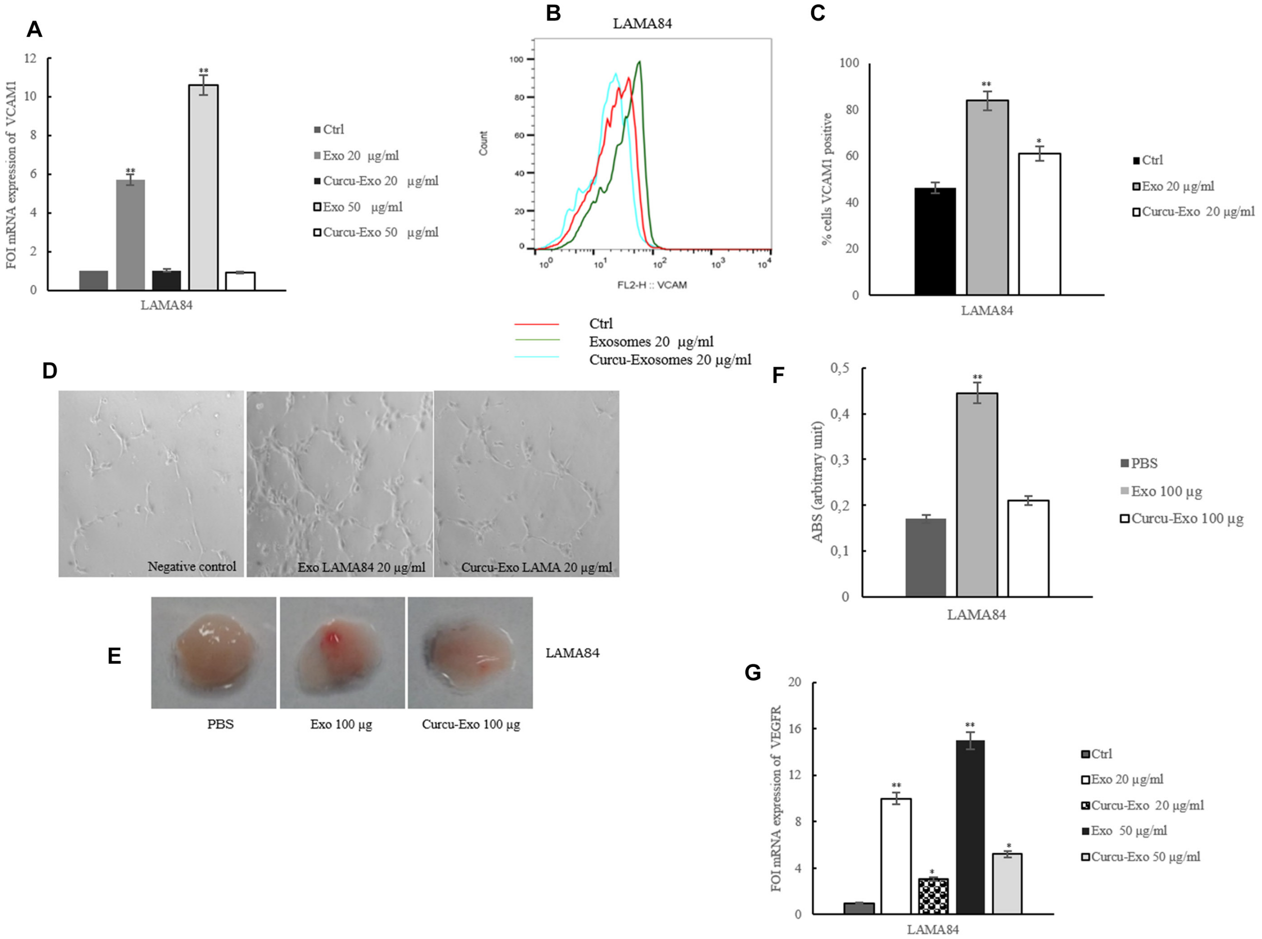

Supplementary Figure S4: Treatment of HUVECs with Curcu-Exosomes modulated VCAM1 expression. (**A**) Real time PCR analysis showed that VCAM1 mRNA expression decreased in dose dependent manner in EC treated with Curcu-exosomes compared to control exosomes. (**B**) FACS analysis showed that VCAM1protein expression decreased in Curcu-exosomes respect to control exosome. (**C**) Histogram shows the percentage of VCAM1 positive HUVECs after 6 hours of treatment with: low serum medium (Ctrl), 20 µg/ml of exosomes (Exo 20 µg/ml) and 20 µg/ml of Curcu-exosomes (Curcu-exo 20 µg/ml). Values are the mean ± SD of 3 fields in three independent experiments $*p \leq 0.05$, $**p \leq 0.01$. (**D**) LAMA84 exosomes stimulate in vitro and in vivo angiogenesis. Phase contrast micrographs showing that LAMA84 control exosomes induce an endothelial network formation on Matrigel, LAMA84 Curcu-exosomes revert this effect. No tube formation is observed when HUVECs are plated in low-serum medium (negative control). (**E**) Matrigel plug containing LAMA84 exosomes stimulate angiogenesis in mice (Exo 100 µg), this effect revert with LAMA84 Curcu-exosomes (Curcu-Exo 100 µg), PBS was used as negative control. (**F**) Haemoglobin concentration in the exosomes-containing Matrigel was evaluated with Dabkin's assay. (**G**) Treatment of HUVECs with Curcu-Exosomes modulated VEGF expression.

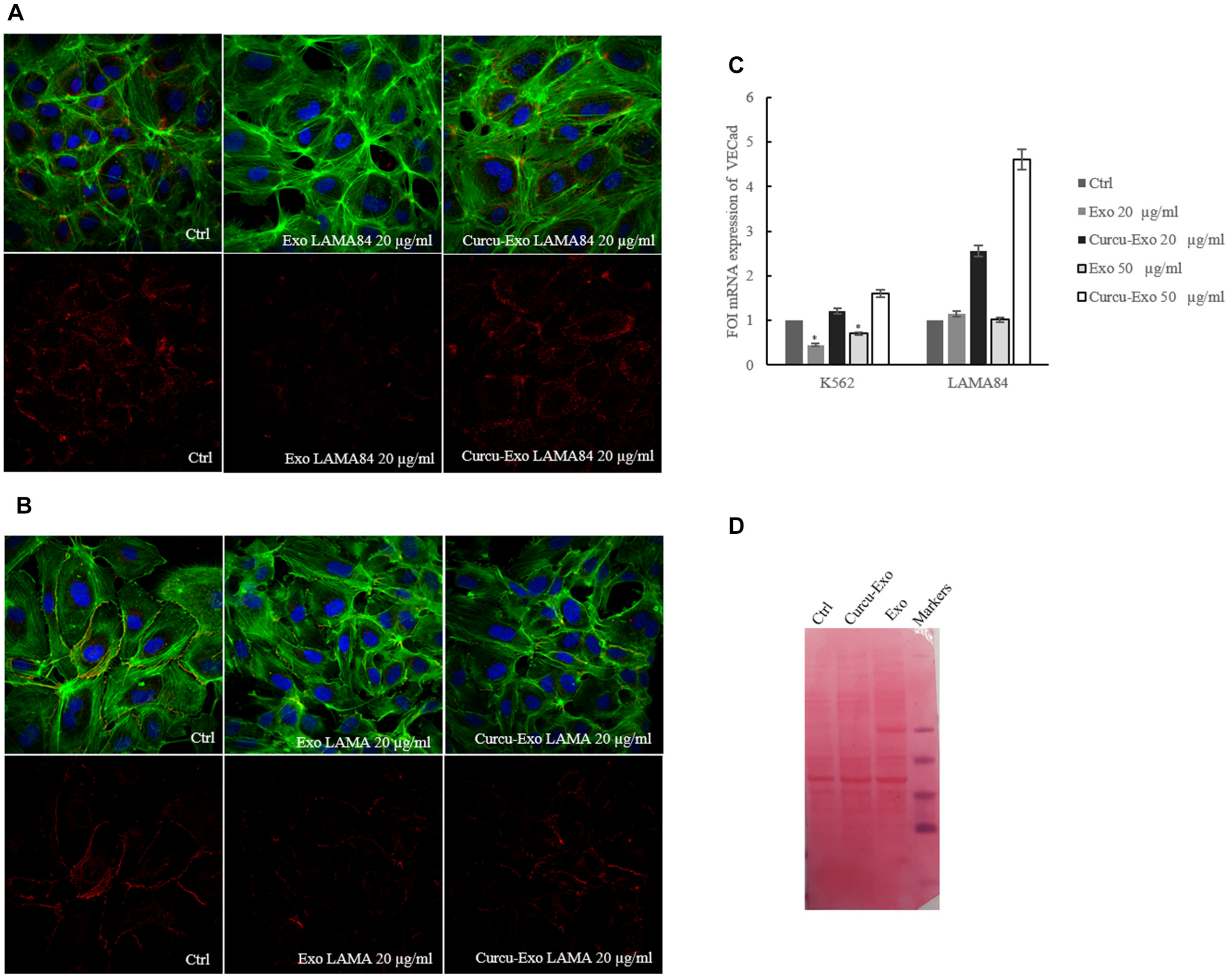

Supplementary Figure S5: Alteration of HUVEC monolayer after addition of LAMA84 exosomes. (**A**) Analysis at confocal microscopy of ZO-1 localization in HUVECs treated with LAMA84 exosomes revealed a decrease of immunostaining compared to untreated cells (control). The treatment with LAMA84 Curcu-exosomes reverted this effect. (**B**) Analysis at confocal microscopy of VE Cadherin localization in HUVECs treated with LAMA84 exosomes revealed a decrease of immunostaining compared to untreated cells (control). The treatment with LAMA84 Curcu-exosomes reverted this effect. (**C**) Treatment of HUVECs with Curcu-Exosomes modulated VE-Cadherin mRNA expression. (**D**) Nitrocellulose membrane of western blotting for MARCKS stains with red ponceau.

Supplementary Table S1: Spectral reference library

Supplementary Table S2: Protein quantification
